# A Population-Based Outcome-Wide Association Study of the Comorbidities and Sequelae Following COVID-19 Infection

**DOI:** 10.1007/s44197-023-00161-w

**Published:** 2023-10-27

**Authors:** Yuyang Zhang, Junhong Li, Lan Feng, Yaxin Luo, Wendu Pang, Ke Qiu, Minzi Mao, Yao Song, Danni Cheng, Yufang Rao, Xinyi Wang, Yao Hu, Zhiye Ying, Xiaobin Pu, Shuyan Lin, Shaohui Huang, Geoffrey Liu, Wei Zhang, Wei Xu, Yu Zhao, Jianjun Ren

**Affiliations:** 1 Department of Otolaryngology-Head & Neck Surgery, West China Hospital, Sichuan University, Chengdu, China; 2 MRC Integrative Epidemiology Unit, University of Bristol, Bristol, UK; 3 Population Health Sciences, Bristol Medical School, University of Bristol, Bristol, UK; 4 West China Biomedical Big Data Center, West China Hospital, Sichuan University, Chengdu, China; 5 Department of Oto-Rhino-Laryngology, Langzhong People’s Hospital, Langzhong, China; 6 Department of Radiation Oncology, Princess Margaret Cancer Centre and University of Toronto, Toronto, Canada; 7 Division of Medical Oncology and Hematology, Department of Medicine, Princess Margaret Cancer Center, University Health Network, University of Toronto, Toronto, Canada; 8 Department of Biostatistics, Princess Margaret Cancer Centre and Dalla Lana School of Public Health, 10-511, 610 University Avenue Toronto, Toronto, ON Canada

**Keywords:** COVID-19, Outcome-wide association study, Comorbidity, Long-term sequelae, Incidence

## Abstract

**Background:**

Immense attention has been given to the outcome of COVID-19 infection. However, comprehensive studies based on large populational cohort with long-term follow-up are still lacking. This study aimed to investigate the risk of various short-term comorbidities (within one month) and long-term sequelae (above one month) after COVID-19 infection.

**Methods:**

In this large prospective cohort study with 14 months follow-up information based on UK biobank, we included 16,776 COVID-19-positive participants and 58,281 COVID-19-negative participants matched for comparison. The risk of each comorbidity and sequela was evaluated by multivariable logistic regression analysis and presented as hazard ratio (HR) and 95% confidence interval (95% CI).

**Results:**

COVID-19-positive individuals had a higher risk of 47 types of comorbidities within one month following COVID-19 infection, especially those who were older, male, overweight/obese, ever-smoked, with more pre-existing comorbidities and hospitalized. About 70.37% of COVID-19 patients with comorbidities had more than one co-occurring comorbidities. Additionally, only 6 high-risk sequelae were observed after one month of COVID-19 infection, and the incidence was relatively low (< 1%).

**Conclusion:**

In addition to long-term sequelae following COVID-19 infection, plenty of comorbidities were observed, especially in patients with older age, male gender, overweight/obese, more pre-existing comorbidities and severe COVID-19, indicating that more attention should be given to these susceptible persons within this period.

**Supplementary Information:**

The online version contains supplementary material available at 10.1007/s44197-023-00161-w.

## Introduction

Over the past 3 years, corona virus disease 2019 (COVID-19) has spread globally and has become a growing public health concern, posing a serious threat to human survival. As of November 2022, over 637 million cases of COVID-19 have been confirmed globally, of which about 1.04% have died [1]. In the context of widespread infection, the possible systemic comorbidities and sequelae of COVID-19 may cause panic.

It has been previously reported that patients with COVID-19 may experience various adverse and persistent symptoms during hospitalization (comorbidity) and after recovery (sequelae or post-acute COVID-19 syndrome), including dyspnea, pain, respiratory diseases, cardiovascular diseases, digestive diseases, neurological disorders, renal failure, diabetes, musculoskeletal diseases, anxiety and depression, etc. [2–16] However, the prevalence and severity of COVID-19-related comorbidities and sequelae remain controversial. Large-scale and long-term cohort studies are still needed to demonstrate the incidence of COVID-19-related comorbidities and sequelae, as well as individual differences in susceptibility, which is of great significance for clinical strategy determination.

The aim of this study is to perform a comprehensive outcome-wide association analysis of COVID-19 comorbidities (short-term) and sequelae (long-term), and individual susceptibilities based on the large prospective UK Biobank (UKB) cohort.

## Material and Methods

### Study Design and Data Source

This prospective population-based outcome-wide association study was conducted in the UKB, which is a national prospective cohort that recruited over 500,000 participants aged 40–69 years from 22 assessment centers across the United Kingdom between 2006 and 2010. UKB obtained ethical approval from the North West Multicenter Research Ethics Committee [17], and has collected extensive detailed baseline and long-term follow-up data, including real-time updated extensive clinical records. SARS-CoV-2 testing results were obtained from Public Health England (PHE), Public Health Scotland (PHS) and Secure Anonymized Information Linkage (SAIL). Hospitalization information of participants from England, Scotland, and Wales were acquired from Hospital Episode Statistics, the Scottish Morbidity Record, and the Patient Episode Database for Wales, respectively. Mortality data were extracted from the National Health Service (NHS) Digital and NHS Central Register.

### Participants and Exposure

A total of 106,760 UKB participants who were tested for SARS-CoV-2 during March 2020 and February 2021 were included. Among the 17,832 participants with at least one positive SARS-CoV-2 test (COVID-19-positve participants), those who were tested with positive result after 2021-2-23 (had limited follow-up time, n = 1041), or withdrew during follow-up (n = 15) were excluded. We further defined the severity of COVID-19 according to the presence of death or hospitalization due to COVID-19. For these 88,928 participants without any positive SARS-CoV-2 test result (COVID-19-negative participants), we excluded those who were reported to die or hospitalize due to COVID-19 (n = 303), died before the pandemic of COVID-19 (2020-1-31, n = 1,095) or withdrew during follow-up (n = 14). Then for each COVID-19-positive participant, we matched up to 4 COVID-19-negative participants according to birth year, sex, and Townsend deprivation index (TDI) by propensity scores. Finally, we excluded COVID-19-negative participants who died before the SARS-CoV-2 test date of their matched COVID-19-positive counterparts. These inclusion and exclusion criteria, and matching process resulted in a final cohort of 16,776 COVID-19-positive participants and 58,281 COVID-19-negative participants (Supplementary Figs. 1–2).

### Outcome and Covariates

Comorbidities and sequelae, defined using 3-digit ICD-10 codes (International Classification of Diseases, 10th revision, excluding codes used for special purposes, injury, poisoning and certain other consequences of external causes, factors influencing health status and contact with health services, as well as external causes of morbidity and mortality) from medical records, referred to newly onset illnesses < 1 month and ≥ 1 month after the diagnosis of COVID-19, respectively [18–20]. We then reclassified the eligible (hazard ratio [HR] > 1, p < 0.05, and case number > 10) diseases into more broadly defined comorbidities and sequelae (Supplementary Tables 1–2). In the case of multiple identical records for the same individual, the date of diagnosis was derived from the earliest record. Ethnicity, body mass index (BMI), smoking status, and Charlson comorbidity index (CCI, without age calculation, Supplementary Table 3) were included as covariates [21–23]. In subgroup analysis, we also defined another covariate, current age, as the age on January 31st, 2020.

### Follow-Up

The start date of follow-up was the test date for COVID-19-positive participants. For COVID-19-negative participants, it was the same as that for their matched counterparts. Follow-up ended on the day of (1) specific disease diagnosis, (2) death, or (3) end of follow-up (March 31st, 2021), whichever came first. The longest follow-up duration was 14 months.

### Statistics Analyses

Student’s t-tests and Chi-square tests were performed as appropriate to assess the differences among groups. Univariable conditional Cox proportional hazards models were performed to identify eligible diseases for comorbidity and sequela reclassification, and outcome-wide association analyses for the risk of COVID-19-related comorbidities and sequelae were conducted using multivariable conditional Cox proportional hazards models adjusting for ethnicity, BMI, smoking status, and CCI [24]. In the specific analysis of each disease, participants diagnosed with corresponding disease before COVID-19 were excluded. In the analysis of death in COVID-19-positive participants, we also excluded individuals who died on the day of diagnosis. To explore the impact of severity of COVID-19 on COVID-19-related sequelae, we additionally adjusted for age, sex, and TDI. In subgroup analyses, participants were stratified by current age (< 65 and ≥ 65 years), sex (female and male), BMI (< 25 kg/m^2^ and ≥ 25 kg/m^2^), smoking status (ever-smoker and never-smoker), and CCI (≤ 1 and ≥ 2).

All analyses were performed using R software (version 3.6.3, https://www.r-project.org/), and a two-tailed p < 0.05 was considered statistically significant.

## Results

### Participant Characteristics

A total of 16,776 COVID-19-positive participants were included and 58,281 COVID-19-negative participants were matched for comparison (1:4). A total of 2670 participants were hospitalized due to COVID-19, and 1169 participants died of COVID-19. Compared with COVID-19-negative participants, COVID-19-positive participants were younger (64.6 vs. 65.8, p < 0.001), more deprived (TDI: − 0.7 ± 3.3 vs. − 0.9 ± 3.3) and more overweight/obese (normal BMI: 25.5% vs 30.1%, p < 0.001, Supplementary Table 4).

### Descriptive Analysis of COVID-19-Related Comorbidity Burdens

**Fig. 1:**
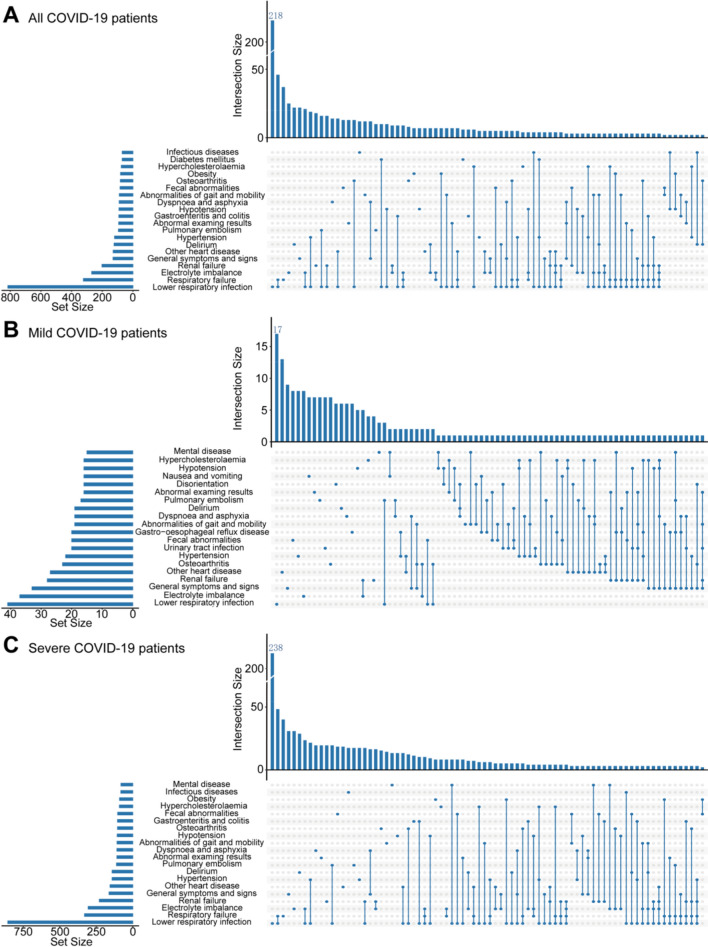
Upset plot representing the coexistence of comorbidities in **A** all COVID-19 patients, **B** mild (non-hospitalized) COVID-19 patients and **C** severe (hospitalized) COVID-19 patients, respectively. *COVID-19* corona virus disease 2019

We observed that compared with COVID-19-negative participants, 121 types of comorbidities showed significantly higher incidences in participants with COVID-19 (HR > 1 and p < 0.05, Supplementary Table 5). In the outcome-wide association analysis adjusting for ethnicity, BMI, CCI and smoking status, 47 out of the 51 reclassified comorbidities showed higher risks in COVID-19-positive participants (HR > 1 and p < 0.05, Table 1). Representative comorbidities included lower respiratory infection (incidence: 5.93%, HR = 48.32, p < 0.001), respiratory failure (incidence: 2.02%, HR = 103.02, p < 0.001), electrolyte imbalance (incidence: 2.00%, HR = 9.41, p < 0.001), renal failure (incidence: 1.51%, HR = 8.8, p < 0.001), hypertension (incidence: 1.25%, HR = 2.49, p < 0.001) and other heart disease (incidence: 1.05%, HR = 3.92, p < 0.001, Table 1). However, the incidence rates of the other remaining 40 types of COVID-19-related comorbidities were less than 1%. Besides, among all COVID-19-positive participants who developed COVID-19-related comorbidities, 70.37% of them were reported to have two or more co-occurring comorbidities, and the most common form of co-occurrence was respiratory failure plus lower respiratory infection (Fig. 1 and Supplementary Fig. 3).

**Table 1:** The incidence and hazard ratio of comorbidity in COVID-19 individuals and matched negative comparisons, adjusted for ethnicity, BMI, smoking status, and CCI

Comorbidity category	Comorbidity	COVID-19 negative			COVID-19 positive			HR (95% CI)	p value
		No. negative participants	No. case	Incidence	No. positive participants	No. case	Incidence		
Infection	Gastroenteritis and colitis	55,405	52	0.09%	15,963	105	0.66%	7.9 (5.32–11.72)	**< 0.001**
	Septicaemia	56,744	29	0.05%	16,187	66	0.41%	16.58 (8.29–33.18)	**< 0.001**
	Infectious diseases	55,400	37	0.07%	15,767	82	0.52%	8.86 (5.61–14)	**< 0.001**
Blood cell disease	Blood cell disease	52,540	92	0.18%	15,184	78	0.51%	2.71 (1.93–3.8)	**< 0.001**
Metabolism	Diabetes mellitus	52,643	66	0.13%	14,897	76	0.51%	3.48 (2.27–5.34)	**< 0.001**
	Hypoglycaemia	57,822	16	0.03%	16,595	36	0.22%	8.32 (3.63–19.09)	**< 0.001**
	Vitamin deficiency	56,977	33	0.06%	16,302	55	0.34%	7.31 (4.41–12.1)	**< 0.001**
	Obesity	52,437	120	0.23%	15,050	91	0.60%	2.33 (1.6–3.39)	**< 0.001**
	Hypercholesterolaemia	48,823	119	0.24%	14,049	92	0.65%	3.11 (2.28–4.25)	**< 0.001**
	Electrolyte imbalance	54,393	137	0.25%	15,314	306	2.00%	9.41 (7.38–12)	**< 0.001**
Nervous system	Dementia	57,836	18	0.03%	16,326	35	0.21%	8.72 (4.38–17.37)	**< 0.001**
	Delirium	57,561	35	0.06%	16,197	142	0.88%	17.31 (10.93–27.42)	**< 0.001**
Mental disease	Mental disease	49,566	118	0.24%	14,401	81	0.56%	2.24 (1.62–3.09)	**< 0.001**
Circulation system	Hypertension	38,523	218	0.57%	11,404	142	1.25%	2.49 (1.91–3.26)	**< 0.001**
	Chronic ischaemic heart disease	53,327	69	0.13%	15,421	44	0.29%	2.23 (1.46–3.39)	**< 0.001**
	Pulmonary embolism	57,289	43	0.08%	16,496	112	0.68%	11.85 (7.68–18.27)	**< 0.001**
	Other heart disease	51,551	162	0.31%	14,803	156	1.05%	3.92 (3.03–5.08)	**< 0.001**
	Vascular disease	56,734	22	0.04%	16,300	24	0.15%	4.92 (2.41–10.03)	**< 0.001**
	Hypotension	56,069	57	0.10%	15,969	107	0.67%	8.17 (5.57–11.97)	**< 0.001**
Respiratory system	Lower respiratory infection	53,400	88	0.16%	14,464	857	5.93%	48.32 (36.02–64.8)	**< 0.001**
	COPD/emphysema	57,048	17	0.03%	16,287	72	0.44%	38.3 (11.59–126.54)	**< 0.001**
	Asthma	51,511	67	0.13%	14,821	46	0.31%	2.29 (1.51–3.49)	**< 0.001**
	Other lung disease	56,192	60	0.11%	16,105	73	0.45%	5.09 (3.42–7.59)	**< 0.001**
	Pleural effusion	56,560	61	0.11%	16,194	50	0.31%	3.79 (2.46–5.84)	**< 0.001**
	Respiratory failure	57,852	17	0.03%	16,358	331	2.02%	103.02 (50.43–210.46)	**< 0.001**
Digestive system	Gastro-oesophageal reflux disease	51,996	105	0.20%	15,210	65	0.43%	2.36 (1.7–3.28)	**< 0.001**
	Diverticular disease of intestine	55,837	63	0.11%	16,110	38	0.24%	2.42 (1.56–3.73)	**< 0.001**
	Fecal abnormalities	53,098	93	0.18%	15,148	104	0.69%	4.1 (3.01–5.6)	**< 0.001**
	Fatty liver	57,178	43	0.08%	16,440	28	0.17%	2.02 (1.16–3.51)	**0.012**
Skin	Cellulitis	56,592	17	0.03%	16,167	12	0.07%	2.94 (1.2–7.24)	**0.019**
	Rash and dermatitis	57,556	10	0.02%	16,540	40	0.24%	17.49 (7.37–41.51)	**< 0.001**
	Decubitus ulcer	57,944	18	0.03%	16,530	66	0.40%	37.43 (14.24–98.38)	**< 0.001**
Musculoskeletal system	Osteoarthritis	52,072	134	0.26%	15,092	105	0.70%	2.78 (2.1–3.68)	**< 0.001**
Genitourinary system	Renal failure	53,961	114	0.21%	15,191	230	1.51%	8.8 (6.69–11.58)	**< 0.001**
	Urinary tract infection	54,434	61	0.11%	15,389	68	0.44%	4.69 (3.16–6.97)	**< 0.001**
	Hyperplasia of prostate	54,705	73	0.13%	15,862	37	0.23%	2.09 (1.34–3.24)	**0.001**
Symptom	Arrhythmia	56,376	42	0.07%	16,213	52	0.32%	5.23 (3.27–8.37)	**< 0.001**
	Cough	57,096	18	0.03%	16,345	74	0.45%	18.47 (9.66–35.29)	**< 0.001**
	Dyspnea and asphyxia	55,959	51	0.09%	16,012	109	0.68%	10.04 (6.6–15.27)	**< 0.001**
	Nausea and vomiting	54,538	64	0.12%	15,772	43	0.27%	2.4 (1.59–3.64)	**< 0.001**
	Abnormalities of gait and mobility	55,922	79	0.14%	15,567	108	0.69%	5.59 (4.01–7.8)	**< 0.001**
	Urinary abnormality	52,228	83	0.16%	14,996	77	0.51%	3.55 (2.52–5.01)	**< 0.001**
	Disorientation	57,407	32	0.06%	16,297	48	0.29%	5.81 (3.5–9.66)	**< 0.001**
	Other cognitive symptoms	57,933	11	0.02%	16,584	15	0.09%	4.93 (1.6–15.2)	**0.005**
	Emotional state symptoms and signs	57,918	13	0.02%	16,615	32	0.19%	11.93 (4.94–28.83)	**< 0.001**
	General symptoms and signs	50,656	124	0.24%	14,433	163	1.13%	5.38 (4.1–7.07)	**< 0.001**
	Abnormal examing results	54,359	94	0.17%	15,616	109	0.70%	4.6 (3.37–6.26)	**< 0.001**

*COVID-19* corona virus disease 2019, *BMI* body mass index, *CCI* Charlson comorbidity index, *HR* hazard ratio, *CI* confidence interval, *COPD* chronic obstructive pulmonary disease. Bold indicates p values less than 0.05

In addition, the mortality of COVID-19-positive participants was significantly higher than COVID-19-negative participants regardless of COVID-19 severity (COVID-19-positive vs. COVID-19-negative: 7.09% vs. 0.91%, HR = 10.6, p < 0.001; mild COVID-19-positive vs. COVID-19-negative: 1.47% vs. 0.80%, HR = 2.11, p < 0.001; severe COVID-19-positive vs. COVID-19-negative: 37.36% vs. 1.43%, HR = 44.15, p < 0.001, Supplementary Table 6).

### Burden of Comorbidities by COVID-19 Severity

Compared with mild COVID-19 patients (non-hospitalized), severe COVID-19 patients (hospitalized) were more likely to be male, older, deprived, obese, ever-smokers and had higher CCI scores (Supplementary Table 7). As expected, they had higher comorbidity burdens (Supplementary Table 8). Compared with COVID-19-negative participants, severe COVID-19 patients had significantly higher risks of having 48/51 types of COVID-19-related comorbidities, among which the incidence rates of lower respiratory infection (61.68%, HR = 12,351.54, p < 0.001), electrolyte imbalance (13.88%, HR = 54.76, p < 0.001), respiratory failure (13.83%, HR = 1394.47, p < 0.001), hypertension (11.04%, HR = 32.55, p < 0.001) and renal failure (10.86%, HR = 81.82, p < 0.001) were relatively higher. Notably, only severe COVID-19 patients were at an increased risk of hearing loss (incidence: 0.59%, HR = 7.57, p = 0.001). In contrast, mild COVID-19 patients only had 11/51 types of comorbidities with incidence rates less than 0.5%: septicaemia (HR = 2.89, p = 0.020), vitamin deficiency (HR = 2.14, p = 0.043), delirium (HR = 3.66, p < 0.001), lower respiratory infection (HR = 2.41, p < 0.001), respiratory failure (HR = 3.32, p = 0.031), rash and dermatitis (HR = 3.31, p = 0.031), decubitus ulcer (HR = 34.6, p = 0.004), cough (HR = 5.55, p = 0.001), dyspnea and asphyxia (HR = 2.36, p = 0.013), disorientation (HR = 2.18, p = 0.033) and emotional state symptoms and signs (HR = 4.68, p = 0.020, Table 2, and Supplementary Fig. 4). Besides, severe COVID-19 patients had more co-occurrence comorbidities, with lower respiratory infection plus respiratory failure being the most common form. On the other hand, the incidence of co-occurring comorbidities in mild COVID-19 patients was relatively lower, and renal failure plus electrolyte imbalance was the commonest (Fig. 1B ~ C and Supplementary Fig. 3B ~ C).

**Table 2:** Clinical comorbidity of COVID-19 individuals with different severity (mild/non-hospitalized and severe/hospitalized) and matched negative comparisons, adjusted for ethnicity, BMI, smoking status, and CCI

Mild COVID-19 patients compared with match negative patients								
Comorbidity	COVID-19 negative			Mild COVID-19			HR (95% CI)	p value
	No. negative participants	No. case	Incidence	No. mild participants	No. case	Incidence		
Septicaemia	47,003	24	0.05%	13,815	13	0.09%	2.89 (1.18–7.1)	**0.020**
Vitamin deficiency	47,161	25	0.05%	13,801	14	0.10%	2.14 (1.02–4.47)	**0.043**
Delirium	47,692	25	0.05%	13,841	19	0.14%	3.66 (1.69–7.9)	**< 0.001**
Lower respiratory infection	44,286	72	0.16%	13,141	41	0.31%	2.41 (1.56–3.71)	**< 0.001**
Respiratory failure	47,900	13	0.03%	14,023	8	0.06%	3.32 (1.11–9.87)	**0.031**
Rash and dermatitis	47,634	10	0.02%	13,966	8	0.06%	3.31 (1.11–9.86)	**0.031**
Decubitus ulcer	47,970	12	0.03%	13,995	8	0.06%	34.6 (3.09–386.8)	**0.004**
Cough	47,277	12	0.03%	13,882	13	0.09%	5.55 (1.96–15.72)	**0.001**
Dyspnea and asphyxia	46,364	38	0.08%	13,688	19	0.14%	2.36 (1.2–4.64)	**0.013**
Disorientation	47,556	27	0.06%	13,863	16	0.12%	2.18 (1.07–4.44)	**0.033**
Emotional state symptoms and signs	47,937	10	0.02%	14,008	7	0.05%	4.68 (1.27–17.21)	**0.020**

Severe COVID-19 patients compared with match negative patients								
Comorbidity	COVID-19 negative			Severe COVID-19			HR (95% CI)	p value
	No. negative participants	No. case	Incidence	No. severe participants	No. case	Incidence		
Gastroenteritis and colitis	9517	9	0.09%	2351	92	3.91%	34.25 (15.75–74.5)	**< 0.001**
Septicaemia	9741	5	0.05%	2372	53	2.23%	125.72 (18.15–870.98)	**< 0.001**
Infectious diseases	9504	9	0.09%	2297	70	3.05%	39.34 (15.4–100.51)	**< 0.001**
Blood cell disease	8970	14	0.16%	2148	63	2.93%	22.71 (9.87–52.25)	**< 0.001**
Diabetes mellitus	8936	10	0.11%	1964	70	3.56%	58 (13.92–241.76)	**< 0.001**
Hypoglycaemia	9971	4	0.04%	2561	28	1.09%	43.95 (7.38–261.8)	**< 0.001**
Vitamin deficiency	9816	8	0.08%	2501	41	1.64%	60.79 (14.23–259.66)	**< 0.001**
Obesity	9032	25	0.28%	2120	81	3.82%	10.33 (5.2–20.54)	**< 0.001**
Hypercholesterolaemia	8114	18	0.22%	1800	76	4.22%	30.55 (12.74–73.27)	**< 0.001**
Electrolyte imbalance	9278	33	0.36%	1938	269	13.88%	54.76 (31.36–95.62)	**< 0.001**
Dementia	9913	6	0.06%	2472	28	1.13%	38.94 (8.66–175.09)	**< 0.001**
Delirium	9869	10	0.10%	2356	123	5.22%	64.26 (25.63–161.14)	**< 0.001**
Mental disease	8632	20	0.23%	2055	66	3.21%	19.96 (8.96–44.49)	**< 0.001**
Sleep apnoea	9739	4	0.04%	2511	15	0.60%	48.03 (2.19–1051.57)	**0.014**
Hearing loss	9704	7	0.07%	2544	15	0.59%	7.57 (2.2–26.08)	**0.001**
Hypertension	6022	40	0.66%	1087	120	11.04%	32.55 (14.59–72.62)	**< 0.001**
Chronic ischaemic heart disease	8947	15	0.17%	2133	37	1.73%	12.34 (5.64–26.96)	**< 0.001**
Pulmonary embolism	9856	11	0.11%	2546	95	3.73%	115.89 (31.87–421.38)	**< 0.001**
Other heart disease	8548	32	0.37%	1859	129	6.94%	24.6 (13.87–43.61)	**< 0.001**
Cerebrovascular diseases	9902	8	0.08%	2510	15	0.60%	8.81 (2.21–35.13)	**0.002**
Vascular disease	9736	6	0.06%	2467	18	0.73%	18.24 (4.16–80.03)	**< 0.001**
Hypotension	9568	11	0.11%	2316	91	3.93%	52.27 (20.41–133.84)	**< 0.001**
Lower respiratory infection	9114	16	0.18%	1323	816	61.68%	12,351.54 (625.03–244084.87)	**< 0.001**
COPD/emphysema	9774	3	0.03%	2364	66	2.79%	231.13 (13.41–3982.69)	**< 0.001**
Asthma	8848	6	0.07%	2154	42	1.95%	62.68 (10.51–374.04)	**< 0.001**
Other lung disease	9622	9	0.09%	2317	63	2.72%	87.14 (20.18–376.29)	**< 0.001**
Pleural effusion	9722	15	0.15%	2407	44	1.83%	28.33 (10.76–74.59)	**< 0.001**
Respiratory failure	9952	4	0.04%	2335	323	13.83%	1394.47 (111.81–17,391.22)	**< 0.001**
Gastro-oesophageal reflux disease	8853	20	0.23%	2246	45	2.00%	16.24 (7.47–35.32)	**< 0.001**
Diverticular disease of intestine	9493	14	0.15%	2438	28	1.15%	11.51 (4.76–27.81)	**< 0.001**
Fecal abnormalities	9038	11	0.12%	2090	84	4.02%	32.05 (14.71–69.81)	**< 0.001**
Fatty liver	9861	8	0.08%	2553	24	0.94%	10.1 (3.05–33.38)	**< 0.001**
Decubitus ulcer	9974	6	0.06%	2535	58	2.29%	88.18 (19.69–394.89)	**< 0.001**
Gout	9815	5	0.05%	2528	17	0.67%	37.8 (4.95–288.47)	**< 0.001**
Osteoarthritis	8757	30	0.34%	2100	82	3.90%	12.1 (7.12–20.57)	**< 0.001**
Renal failure	9122	26	0.29%	1860	202	10.86%	81.82 (36.09–185.49)	**< 0.001**
Urinary tract infection	9263	9	0.10%	2146	48	2.24%	41.31 (12.01–142.07)	**< 0.001**
Hyperplasia of prostate	9254	16	0.17%	2348	33	1.41%	16.64 (6.56–42.19)	**< 0.001**
Arrhythmia	9657	8	0.08%	2449	46	1.88%	53.75 (14.26–202.67)	**< 0.001**
Cough	9819	6	0.06%	2463	61	2.48%	74.81 (17.01–329.06)	**< 0.001**
Dyspnea and asphyxia	9595	13	0.14%	2324	90	3.87%	38.23 (17.61–83.02)	**< 0.001**
Nausea and vomiting	9363	7	0.07%	2341	27	1.15%	32.74 (7.91–135.54)	**< 0.001**
Abnormalities of gait and mobility	9531	15	0.16%	2096	89	4.25%	40.7 (17.99–92.08)	**< 0.001**
Urinary abnormality	8854	13	0.15%	2088	64	3.07%	23.8 (10.74–52.71)	**< 0.001**
Disorientation	9851	5	0.05%	2434	32	1.31%	48.14 (10.48–221.15)	**< 0.001**
Emotional state symptoms and signs	9981	3	0.03%	2607	25	0.96%	68.56 (7.5–626.41)	**< 0.001**
General symptoms and signs	8635	24	0.28%	1893	130	6.87%	32.96 (17.53–62)	**< 0.001**
Abnormal examing results	9281	11	0.12%	2233	93	4.16%	50.78 (19.97–129.11)	**< 0.001**

*COVID-19* corona virus disease 2019, *BMI* body mass index, *CCI* Charlson comorbidity index, *HR* hazard ratio, *CI* confidence interval, *COPD* chronic obstructive pulmonary disease. Bold indicates p values less than 0.05

### Subgroup Analyses of COVID-19-Related Comorbidities by Age, Sex, BMI, Smoking Status and CCI

**Fig. 2:**
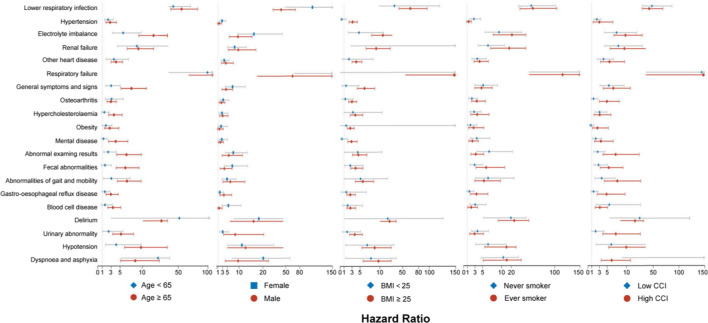
Differences in adjusted hazards ratio of clinical comorbidities among COVID-19-positive participants compared with COVID-19-negative participants stratified by age, sex, BMI, smoking status, and CCI, respectively. Note: Only the top 20 comorbidities in terms of incidence were shown. Analyses were adjusted for ethnicity, BMI, smoking status and CCI as appropriate. *COVID-19* corona virus disease 2019, *BMI* body mass index, *CCI* Charlson comorbidity index

To understand whether the burden of COVID-19-related comorbidities differed among various populations, we further carried out subgroup analyses stratified by current age, sex, BMI, smoking status and CCI, respectively (Fig. 2).

In terms of subgroup analysis for current age, older COVID-19-positive participants (> 65 years) were at a significantly higher risk of 48/51 clinical comorbidities, whereas only 26/51 comorbidities were significant in younger COVID-19 patients (Table 3). Specifically, among younger participants, COVID-19-positive individuals had a higher risk of respiratory failure (HR = 99.05, p < 0.001), vitamin deficiency (HR = 66.51, p = 0.004), delirium (HR = 51.65, p = 0.006), septicaemia (HR = 50.86, p = 0.026), etc. However, the incidences of these comorbidities were less than 1%, except for respiratory failure (incidence: 1.23%). In contrast, 16 out of the 48 comorbidities in elderly COVID-19-positive patients had an incidence greater than 1%, e.g., lower respiratory infection (9.22%), electrolyte imbalance (3.72%), respiratory failure (3.00%), renal failure (2.79%), etc.

**Table 3:** Clinical comorbidity in COVID-19 individuals and matched negative comparisons by age, adjusted for ethnicity, BMI, smoking status, and CCI

Comorbidity	Older								Younger							
	COVID-19 negative			COVID-19 positive			HR (95% CI)	p value	COVID-19 negative			COVID-19 positive			HR (95% CI)	p value
	No. negative participants	No. case	Incidence	No. positive participants	No. case	Incidence			No. negative participants	No. case	Incidence	No. positive participants	No. case	Incidence		
Gastroenteritis and colitis	29,129	34	0.12%	7101	75	1.06%	10.02 (5.92–16.94)	**< 0.001**	26,276	18	0.07%	8862	30	0.34%	10.87 (3.65–32.33)	**< 0.001**
Septicaemia	29,829	21	0.07%	7184	54	0.75%	17.78 (8.01–39.47)	**< 0.001**	26,915	8	0.03%	9003	12	0.13%	50.86 (1.59–1627.2)	**0.026**
Infectious diseases	29,009	22	0.08%	6912	71	1.03%	12.45 (7.13–21.74)	**< 0.001**	26,391	15	0.06%	8855	11	0.12%	2.16 (0.61–7.68)	0.234
Blood cell disease	27,108	60	0.22%	6598	59	0.89%	3.41 (2.25–5.18)	**< 0.001**	25,432	32	0.13%	8586	19	0.22%	1.62 (0.8–3.28)	0.177
Diabetes mellitus	27,037	44	0.16%	6341	33	0.52%	2.63 (1.47–4.7)	**0.001**	25,606	22	0.09%	8556	43	0.50%	16.14 (3.99–65.28)	**< 0.001**
Hypoglycaemia	30,615	10	0.03%	7496	31	0.41%	12.87 (4.59–36.04)	**< 0.001**	27,207	6	0.02%	9099	5	0.05%	1.98 (0.31–12.73)	0.472
Vitamin deficiency	30,058	26	0.09%	7301	36	0.49%	6.08 (3.39–10.89)	**< 0.001**	26,919	7	0.03%	9001	19	0.21%	66.51 (3.7–1196.32)	**0.004**
Obesity	27,571	58	0.21%	6704	41	0.61%	2.69 (1.52–4.75)	**< 0.001**	24,866	62	0.25%	8346	50	0.60%	1.83 (1.05–3.19)	**0.033**
Hypercholesterolaemia	23,725	73	0.31%	5633	61	1.08%	3.63 (2.41–5.48)	**< 0.001**	25,098	46	0.18%	8416	31	0.37%	1.46 (0.82–2.61)	0.196
Electrolyte imbalance	28,114	94	0.33%	6482	241	3.72%	12.52 (9.2–17.03)	**< 0.001**	26,279	43	0.16%	8832	65	0.74%	5.69 (3.32–9.74)	**< 0.001**
Dementia	30,489	16	0.05%	7223	32	0.44%	10.5 (4.8–22.96)	**< 0.001**	27,347	2	0.01%	9103	3	0.03%	NA	1.000
Delirium	30,278	32	0.11%	7118	124	1.74%	16.5 (10.17–26.78)	**< 0.001**	27,283	3	0.01%	9079	18	0.20%	51.65 (3.03–881.71)	**0.006**
Mental disease	26,429	48	0.18%	6352	46	0.72%	4.03 (2.41–6.74)	**< 0.001**	23,137	70	0.30%	8049	35	0.43%	1.3 (0.8–2.1)	0.290
Hypertension	16,708	128	0.77%	3876	79	2.04%	2.77 (1.83–4.19)	**< 0.001**	21,815	90	0.41%	7528	63	0.84%	2.32 (1.54–3.49)	**< 0.001**
Chronic ischaemic heart disease	26,991	46	0.17%	6553	35	0.53%	2.69 (1.58–4.56)	**< 0.001**	26,336	23	0.09%	8868	9	0.10%	1.37 (0.57–3.32)	0.485
Pulmonary embolism	30,197	34	0.11%	7444	71	0.95%	10.38 (6.15–17.53)	**< 0.001**	27,092	9	0.03%	9052	41	0.45%	16 (5.96–42.93)	**< 0.001**
Other heart disease	25,562	123	0.48%	6014	120	2.00%	4.09 (3–5.56)	**< 0.001**	25,989	39	0.15%	8789	36	0.41%	3.62 (1.9–6.91)	**< 0.001**
Cerebrovascular diseases	30,329	30	0.10%	7344	16	0.22%	2.36 (1.1–5.07)	**0.027**	27,266	9	0.03%	9090	3	0.03%	0.66 (0.11–3.99)	0.652
Vascular disease	29,746	18	0.06%	7280	18	0.25%	3.88 (1.71–8.81)	**0.001**	26,988	4	0.01%	9020	6	0.07%	NA	0.999
Hypotension	29,312	41	0.14%	6971	87	1.25%	9.69 (6.02–15.6)	**< 0.001**	26,757	16	0.06%	8998	20	0.22%	4.12 (1.74–9.78)	**0.001**
Lower respiratory infection	27,521	60	0.22%	5919	546	9.22%	55.53 (37.33–82.62)	**< 0.001**	25,879	28	0.11%	8545	311	3.64%	41.46 (24.26–70.85)	**< 0.001**
COPD/emphysema	29,927	15	0.05%	7214	62	0.86%	48.85 (11.15–214.1)	**< 0.001**	27,121	2	0.01%	9073	10	0.11%	NA	0.998
Asthma	27,184	30	0.11%	6599	19	0.29%	3.03 (1.43–6.42)	**0.004**	24,327	37	0.15%	8222	27	0.33%	2.3 (1.25–4.22)	**0.007**
Other lung disease	29,347	44	0.15%	7102	56	0.79%	5.33 (3.28–8.66)	**< 0.001**	26,845	16	0.06%	9003	17	0.19%	4.74 (1.71–13.15)	**0.003**
Pleural effusion	29,628	44	0.15%	7188	39	0.54%	5.04 (2.96–8.57)	**< 0.001**	26,932	17	0.06%	9006	11	0.12%	2.26 (0.81–6.28)	0.117
Respiratory failure	30,588	10	0.03%	7339	220	3.00%	361.26 (67.59–1930.85)	**< 0.001**	27,264	7	0.03%	9019	111	1.23%	99.05 (22.6–434.2)	**< 0.001**
Gastro-oesophageal reflux disease	26,893	60	0.22%	6679	40	0.60%	2.94 (1.87–4.62)	**< 0.001**	25,103	45	0.18%	8531	25	0.29%	1.58 (0.92–2.74)	0.100
Diverticular disease of intestine	29,018	53	0.18%	7131	30	0.42%	2.49 (1.52–4.08)	**< 0.001**	26,819	10	0.04%	8979	8	0.09%	18.7 (1.06–329.46)	**0.045**
Fecal abnormalities	27,499	57	0.21%	6506	84	1.29%	6.19 (4.15–9.24)	**< 0.001**	25,599	36	0.14%	8642	20	0.23%	1.63 (0.86–3.1)	0.133
Fatty liver	30,322	22	0.07%	7453	17	0.23%	2.39 (1.12–5.12)	**0.025**	26,856	21	0.08%	8987	11	0.12%	1.45 (0.55–3.83)	0.456
Rash and dermatitis	30,467	7	0.02%	7474	32	0.43%	19.29 (7.04–52.87)	**< 0.001**	27,089	3	0.01%	9066	8	0.09%	7.14 (0.71–71.94)	0.095
Decubitus ulcer	30,634	14	0.05%	7430	54	0.73%	43.89 (14.16–136.01)	**< 0.001**	27,310	4	0.01%	9100	12	0.13%	NA	0.998
Gout	30,078	21	0.07%	7393	17	0.23%	2.66 (1.23–5.73)	**0.013**	27,128	12	0.04%	9046	2	0.02%	0.61 (0.08–4.69)	0.636
Osteoarthritis	26,348	101	0.38%	6360	73	1.15%	2.94 (2.07–4.16)	**< 0.001**	25,724	33	0.13%	8732	32	0.37%	3.11 (1.68–5.73)	**< 0.001**
Renal failure	27,530	92	0.33%	6340	177	2.79%	9.11 (6.61–12.55)	**< 0.001**	26,431	22	0.08%	8851	53	0.60%	8.78 (4.42–17.47)	**< 0.001**
Urinary tract infection	28,214	46	0.16%	6592	59	0.90%	5.6 (3.54–8.87)	**< 0.001**	26,220	15	0.06%	8797	9	0.10%	2.14 (0.75–6.06)	0.154
Hyperplasia of prostate	27,921	54	0.19%	6880	28	0.41%	2.71 (1.58–4.65)	**< 0.001**	26,784	19	0.07%	8982	9	0.10%	2.48 (0.88–7.03)	0.087
Arrhythmia	29,571	28	0.09%	7245	29	0.40%	6.12 (3.06–12.24)	**< 0.001**	26,805	14	0.05%	8968	23	0.26%	7.67 (2.82–20.85)	**< 0.001**
Cough	30,146	9	0.03%	7353	50	0.68%	29.66 (11.06–79.53)	**< 0.001**	26,950	9	0.03%	8992	24	0.27%	8.86 (3.25–24.2)	**< 0.001**
Dyspnea and asphyxia	29,412	35	0.12%	7134	69	0.97%	8.41 (5.11–13.83)	**< 0.001**	26,547	16	0.06%	8878	40	0.45%	13.53 (5.15–35.56)	**< 0.001**
Nausea and vomiting	28,717	37	0.13%	7050	26	0.37%	2.8 (1.6–4.89)	**< 0.001**	25,821	27	0.10%	8722	17	0.19%	2.15 (1.08–4.26)	**0.029**
Abnormalities of gait and mobility	29,049	59	0.20%	6574	92	1.40%	6.55 (4.42–9.71)	**< 0.001**	26,873	20	0.07%	8993	16	0.18%	3.05 (1.28–7.27)	**0.012**
Urinary abnormality	26,699	58	0.22%	6353	62	0.98%	5.21 (3.37–8.06)	**< 0.001**	25,529	25	0.10%	8643	15	0.17%	2.44 (1.05–5.66)	**0.038**
Disorientation	30,224	23	0.08%	7225	43	0.60%	10.34 (5.32–20.09)	**< 0.001**	27,183	9	0.03%	9072	5	0.06%	0.7 (0.14–3.46)	0.665
Other cognitive symptoms	30,611	8	0.03%	7478	15	0.20%	11.61 (2.37–56.98)	**0.003**	27,322	3	0.01%	9106	0	0.00%	NA	1.000
Emotional state symptoms and signs	30,694	8	0.03%	7527	22	0.29%	15.63 (5.1–47.86)	**< 0.001**	27,224	5	0.02%	9088	10	0.11%	NA	0.998
General symptoms and signs	26,226	82	0.31%	6120	122	1.99%	7.55 (5.21–10.95)	**< 0.001**	24,430	42	0.17%	8313	41	0.49%	3.02 (1.8–5.06)	**< 0.001**
Abnormal examing results	28,158	61	0.22%	6803	81	1.19%	6.45 (4.25–9.78)	**< 0.001**	26,201	33	0.13%	8813	28	0.32%	2.34 (1.34–4.08)	**0.003**

*COVID-19* corona virus disease 2019, *BMI* body mass index, *CCI* Charlson comorbidity index, *HR* hazard ratio, *CI* confidence interval, *COPD* chronic obstructive pulmonary disease, *NA* not available/applicable. Bold indicates p values less than 0.05

Females had higher risks of 36/51 types of COVID-19-related comorbidities, whereas males were at higher risks of having 37/51 types of COVID-19-related comorbidities (Supplementary Table 9). Notably, blood cell disease (incidence: 0.65%, HR = 5.42, p < 0.001), obesity (incidence: 0.61%, HR = 2.34, p = 0.028), hypertension (incidence: 1.95%, HR = 2.77, p < 0.001), cerebrovascular diseases (incidence: 0.13%, HR = 4.74, p = 0.027), vascular disease (incidence: 0.20%, HR = 3.53, p = 0.042), COPD/emphysema (incidence: 0.64%, HR = 1732.86, p = 0.030) and rash and dermatitis (incidence: 0.31%, HR = 33.8, p = 0.006) were observed as significant comorbidities only in males but not in females.

In addition, individuals who were overweight/obese, ever-smoker, or with more comorbidities at baseline (CCI score ≥ 2) had more COVID-19-related comorbidities than their counterparts, respectively (Supplementary Tables 10–12).

### Descriptive Analysis of COVID-19-Related Sequelae Burden

We observed that 14 types of sequelae were positively associated with the infection of COVID-19 (HR > 1 and p < 0.05, Supplementary Table 13). In the outcome-wide association analysis adjusting for ethnicity, BMI, CCI and smoking status, only 6 out of the 11 reclassified more broadly defined sequelae were observed in COVID-19-positive participants, including lower respiratory infection (incidence: 0.10%, HR = 8.33, p < 0.001), immobility (incidence: 0.10%, HR = 4.82, p = 0.001), interstitial pulmonary disease (incidence: 0.12%, HR = 2.4, p = 0.018), fecal abnormalities (incidence: 0.13%, HR = 2.24, p = 0.011), decubitus ulcer (incidence: 0.17%, HR = 1.96, p = 0.020) and urinary incontinence (incidence: 0.21%, HR = 1.81, p = 0.010, Supplementary Table 14), etc. The incidence rates of all these COVID-19-related sequelae were less than 1%.

## Discussion

In this prospective cohort study, we conducted comprehensive outcome-wide association analyses to identify COVID-19-related comorbidities and sequelae in a large population. Overall, 47 types of COVID-19 related comorbidities that occurred within one month after COVID-19 infection were identified, by incidence from high to low, including lower respiratory infection, respiratory failure, electrolyte imbalance, renal failure, hypertension and other heart disease, etc. We also observed that COVID-19-related comorbidities tended to co-occur, especially in severe COVID-19 patients. Besides, older age, male gender, obese/overweight, smoking history, higher CCI scores and severe COVID-19 were risk factors for experiencing more types of comorbidities. Meanwhile, we identified 6 types of COVID-19 related sequelae that began to appear after one month following COVID-19 infection, such as lower respiratory infection, immobility, interstitial pulmonary disease, fecal abnormalities, decubitus ulcer and urinary incontinence. Nonetheless, the incidence rates of these COVID-19-related sequelae were all relatively low (< 1%). Therefore, the public should be urged not to worry too much about these low-morbidities and low-seriousness sequelae.

Previous studies showed that COVID-19 patients had a higher risk of having sequelae, such as myalgia, sexual dysfunction, hearing loss and disturbances of smell and taste [25–28], however, these sequelae were not prominent in our results, as we only observed 6 types of COVID-19-related sequelae with low incidences. However, we observed plenty of comorbidities involving multiple organs after COVID-19 infection, such as respiratory, neurological, circulatory and urinary systems. Besides, the majority of COVID-19 patients developing comorbidities showed more than one comorbidity. Thus, comorbidity was a more prominent issue for COVID-19 patients.

It has been established that SARS-CoV-2 can upregulate the expression of the type 2 angiotensin converting enzyme (ACE-2), and can bind ACE-2 receptors on the surface of the host cells for cell entrance in many organs, which may explain the comorbidities we observed in COVID-19 patients, such as hypertension, diabetes and COPD [29–31]. COVID-19 is associated with a high inflammatory burden and SARS-CoV-2 can affect the myocardium and cardiac biomarker level and lead to myocarditis and heart failure [32–35]. Moreover, interleukin-mediated modulation of phosphokinases and phosphatases, NOD-like receptor thermal protein domain associated protein 3 (NLRP3) inflammasome-mediated inflammation and pathological accumulation of amyloid-β are associated with COVID-19 related neurological disorder. Several studies have found abnormalities in brain structures in COVID-19 patients, such as reduced grey matter thickness, tissue-contrast in the cortex and gyrus, and reduced overall brain size [36–39]. In addition, the activation of the RAS, hemodynamic changes and secondary infection of the urinary tract following COVID-19 infection are associated with the comorbidities and sequelae of urinary system in COVID-19 patients [40–42]. Hearing loss was found in the comorbidities of severe COVID-19 patients, which may be associated with brainstem dysfunction resulting from neuroinflammatory mechanisms. Cytokine storm after COVID-19 infection could damage the auditory glial cells and might play a role in hearing loss [43].

We observed that participants with advanced age, male sex, smoking status or excessive obesity were at higher risks of COVID-19-related comorbidities. Old and obese participants were usually characterized by more pre-existing comorbidities, weaker immune defense, and higher levels of proinflammatory cytokines, which may contribute to their more comorbidities [44, 45]. Besides, the discrepancy of COVID-19 related outcomes between male and female participants could be attributed to the differences in sex hormones, expression levels of ACE2 and Transmembrane protease serine 2 (TMPRSS2), and lifestyles [46]. Moreover, smoking is associated with a higher expression level of ACE2 in airway epithelial cells, which may induce the occurrence of COVID-19 related comorbidities and sequelae [47]. These populations should be paid special attention as they were more susceptible to COVID-19-related comorbidities.

Although the advent of our study provided new insight into the comorbidities and sequelae of COVID-19 patients, a few limitations still existed. First, due to the data limitation, we defined the severity of COVID-19 according to the hospitalization status or death cause, which may lead to partial bias but was acceptable [48]. Second, due to the limited data on COVID-19 medications, we could not assess the effect of COVID-19 medications on COVID-19-related outcomes. Third, our observations were mainly of comorbidities and sequelae associated with the SARS-CoV-2 Alpha variant, which was the main strain in the UK between January 31, 2020 and March 31, 2021, but not the Delta and Omicron variants of SARS-CoV-2, which began to emerged and spread in the UK from March and October 2021, respectively. The SARS-CoV-2 Alpha variant was considered relatively more pathogenic but less infectious than Delta and Omicron variant, therefore, the incidence and severity of sequelae of Delta and Omicron variants might be lower than, also be different from, those of Alpha variants [49–52]. Unfortunately, to date, we are unable to obtain the lagging comorbidities and sequelae data of Delta and Omicron variant for analysis.

In conclusion, 47 types of high-risk comorbidities might occur within one month after COVID-19 infection, especially in patients with older age, overweight/obese, more pre-existing comorbidities and severe COVID-19. And only 6 types of COVID-19-related sequelae appeared after one month following COVID-19 infection, indicating that more attention and health care should be given to these susceptible populations after COVID-19 infection.

### Supplementary Information

Below is the link to the electronic supplementary material.

Supplementary file1 (PDF 937 KB)

## Data Availability

UKB data are available in a public, open access repository. This research has been conducted using the UKB Resource under Application Number 80787 and 69718. The UKB data are available on application to the UKB (http://www.ukbiobank.ac.uk/).

## Abbreviations

COVID-19: Corona virus disease 2019
HR: Hazard ratio
95% CI: 95% confidence interval
UKB: UK Biobank
PHE: Public Health England
PHS: Public Health Scotland
SAIL: Secure Anonymized Information Linkage
NHS: National Health Service
TDI: Townsend deprivation index
ICD: International Classification of Diseases
BMI: Body mass index
CCI: Charlson comorbidity index
COPD: Chronic obstructive pulmonary disease
ACE-2: Type 2 angiotensin converting enzyme
NLRP3: NOD-like receptor thermal protein domain associated protein 3
TMPRSS2: Transmembrane protease serine 2
SARS-CoV-2: Severe acute respiratory syndrome coronavirus 2
