# Supplementary material for: A Population-Based Outcome-Wide Association Study of the Comorbidities and Sequelae Following COVID-19 Infection
Source: J Epidemiol Glob Health. 2023 Oct 27;13(4):870–85. doi: 10.1007/s44197-023-00161-w (PMC10686900; doi:10.1007/s44197-023-00161-w)
Supplement: Supplementary file 1 — Supplementary file1 (PDF 937 KB) [file 44197_2023_161_MOESM1_ESM.pdf]

Supplementary Information

A population-based outcome-wide association study of the comorbidities and sequelae following COVID-19 infection

Yuyang Zhang[1]#, Junhong Li[1]#, Lan Feng [1]#, Yaxin Luo[2], Wendu Pang[1], Ke Qiu[1], Minzi Mao[1], Yao Song[1], Danni Cheng[1], Yufang Rao[1], Xinyi Wang[1], Yao Hu[3], Zhiye Ying[3], Xiaobin Pu[4], Shuyan Lin[1], Shaohui Huang[5], Geoffrey Liu[6], Wei Zhang[3], Wei Xu[7], Yu Zhao[1,3]*, Jianjun Ren[1,3,4]**

[1] *Department of Otolaryngology-Head & Neck Surgery, West China Hospital, Sichuan University, Chengdu, China*

[2] *Department of Epidemiology and Biostatistics, West China School of Public Health and West China Fourth Hospital, Sichuan University, Chengdu, China*

[3] *West China Biomedical Big Data Center, West China Hospital, Sichuan University, Chengdu, China*

[4] *Department of Oto-Rhino-Laryngology, Langzhong People's Hospital, Langzhong, China*

[5] *Department of Radiation Oncology, Princess Margaret Cancer Centre and University of Toronto, Toronto, Canada*

[6] *Department of Medicine, Division of Medical Oncology and Hematology, Princess Margaret Cancer Center, University Health Network, University of Toronto, Toronto, Canada*

[7] *Department of Biostatistics, Princess Margaret Cancer Centre and Dalla Lana School of Public Health, Toronto, Ontario, Canada*

#These authors contributed equally to this work

**** Corresponding author:***

Jianjun Ren, Department of Otolaryngology-Head & Neck Surgery, and West China Biomedical Big Data Center, West China Hospital, Sichuan University, Chengdu, China; Email: Jianjun.Ren@scu.edu.cn

Wei Xu, Department of Biostatistics, Princess Margaret Cancer Centre, 10-511, 610 University Avenue Toronto, Toronto, Ontario, Canada; Email: Wei.Xu@uhnresearch.ca

Yu Zhao, Department of Otolaryngology-Head & Neck Surgery, West China Hospital, Sichuan University, Chengdu, China, Email: yuzhao@wchscu.edu.cn

Table of contents

Supplementary Table 1: Comorbidity categories and corresponding ICD-10 codes. 3
Supplementary Table 2: Sequela categories and corresponding ICD-10 codes. 4
Supplementary Table 3: ICD-10 codes and Charlson Comorbidity Index (CCI) scores. 5
Supplementary Table 4: Characteristics of COVID-19-positive and negative patients. 6
Supplementary Table 5: The outcome-wide association analyses evaluating the hazards ratio (HR) for comorbidities (defined by three-digit ICD-10 codes) of COVID-19-positive participants compared to matched COVID-19-negative individuals. 7
Supplementary Table 6: Adjusted risk of death for COVID-19-positive participants compared with matched COVID-19-negative comparisons. 9
Supplementary Table 7: Characteristics of mild (non-hospitalized) and severe (hospitalized) COVID-19 patients. 10
Supplementary Table 8: Adjusted hazards ratios (HRs) of clinical comorbidities for severe COVID-19 patients (hospitalized) compared with mild COVID-19 patients (non-hospitalized). 11
Supplementary Table 9: The adjusted risk of clinical comorbidity in COVID-19-positive participants compared with matched COVID-19-negative participants in females and males, respectively. 12
Supplementary Table 10: The adjusted risk of clinical comorbidity in COVID-19-positive participants compared with matched COVID-19-negative participants stratified by BMI. 13
Supplementary Table 11: The adjusted risk of clinical comorbidity in COVID-19-positive participants compared with matched COVID-19-negative participants stratified by smoking status. 14
Supplementary Table 12: The adjusted risk of clinical comorbidity in COVID-19-positive participants compared with matched COVID-19-negative participants stratified by CCI score. 15
Supplementary Table 13: The outcome-wide association analyses evaluating the hazards ratio (HR) for sequelae (defined by three-digit ICD-10 codes) of COVID-19-positive participants compared to matched COVID-19-negative participants. 16
Supplementary Table 14: Adjusted hazards ratios (HRs) of sequelae of COVID-19-positive participants compared with matched COVID-19-negative participants. 17
Supplementary Figure 1: Flowchart of the study design. 18
Supplementary Figure 2: The absolute standardized mean difference in all patients and matched patients. 19
Supplementary Figure 3: The distribution of (A) all, (B) mild (non-hospitalized), and (C) severe (hospitalized) COVID-19 patients with different numbers of comorbidities, respectively. 20
Supplementary Figure 4: The proportion of COVID-19-positive participants with different comorbidities, stratified by age, sex, BMI, smoking status, CCI and severity, respectively. 21

Supplementary Table 1: Comorbidity categories and corresponding ICD-10 codes.

Comorbidity category	ICD-10 codes
Gastroenteritis and colitis	A099
Septicaemia	A415, A419
Infectious diseases	B370, B956, B962, B968
Blood cell disease	D509, D649, D696
Diabetes mellitus	E119
Hypoglycaemia	E162
Vitamin deficiency	E538, E559
Obesity	E668, E669
Hypercholesterolaemia	E780
Electrolyte imbalance	E833, E834, E835, E86, E870, E871, E872, E873, E875, E876, E877
Dementia	F019, F03
Delirium	F050, F051, F058, F059
Mental disease	F171, F329, F419
Sleep apnoea	G473
Hearing loss	H919
Hypertension	I10
Chronic ischaemic heart disease	I252, I259
Pulmonary embolism	I269
Other heart disease	I340, I350, I447, I451, I480, I489, I500, I509, I517, I518
Cerebrovascular diseases	I678
Vascular disease	I739, I802
Hypotension	I951, I959
Lower respiratory infection	J128, J181, J189, J22, J690
COPD/Emphysema	J439, J440
Asthma	J459
Other lung disease	J47, J841, J981
Pleural effusion	J90
Respiratory failure	J9600, J9690, J9691
Gastro-oesophageal reflux disease	K219
Diverticular disease of intestine	K579
Fecal abnormalities	K590, R15, R195
Fatty liver	K760
Cellulitis	L031
Rash and dermatitis	L248, R21
Decubitus ulcer	L890, L891, L899
Gout	M109
Osteoarthritis	M199, M549, M819
Renal failure	N179, N183, N189
Urinary tract infection	N390
Hyperplasia of prostate	N40
Arrhythmia	R000, R001
Cough	R05
Dyspnea and asphyxia	R060, R090
Nausea and vomiting	R11
Abnormalities of gait and mobility	R263, R268, R296
Urinary abnormality	R31, R32, R33
Disorientation	R410
Other cognitive symptoms	R418
Emotional state symptoms and signs	R451, R458
General symptoms and signs	R509, R51, R53, R55, R568, R600, R630, R638
Abnormal examing results	R798, R91, R945

ICD-10: international classification of disease, 10th revision.

Supplementary Table 2: Sequela categories and corresponding ICD-10 codes.

**Sequelae category**	**ICD-10 codes**
Septicaemia	A419
Infectious diseases	B956, B968
Delirium	F050, F051
Lower respiratory infection	J128, J189
Interstitial pulmonary diseases	J841
Respiratory failure	J9691
Decubitus ulcer	L891
Fecal abnormalities	R15
Immobility	R263
Urinary abnormality	R32
Emotional state symptoms and signs	R458

ICD-10: international classification of disease, 10th revision.

Supplementary Table 3: ICD-10 codes and Charlson Comorbidity Index (CCI) scores.

Comorbidities	Score	ICD-10 codes
**Age of the patient**		
<50 years	0	/
50-59 years	1	/
60-69 years	2	/
70-79 years	3	/
80 years or more	4	/
**Myocardial infarction**		
Yes	1	I21, I22, I252
No	0	
**Congestive heart failure**		
Yes	1	I50
No	0	
**Peripheral vascular disease**		
Yes	1	I71, I790, I739, R02, Z958, Z959
No	0	
**Cerebrovascular disease**		
Yes	1	G450, G451, G452, G454, G458, G459, G46, I60, I61, I62, I63, I65, I66, I64, I670, I671, I672, I674, I675, I676, I677, I678, I679, I681, I682, I688, I69
No	0	
**Dementia**		
Yes	1	F00, F01, F02, F051
No	0	
**Chronic pulmonary disease**		
Yes	1	J40, J41, J42, J43, J44, J45, J46, J47, J67, J60, J61, J62, J63, J66, J64, J65
No	0	
**Rheumatologic disease**		
Yes	1	M32, M34, M332, M053, M058, M059, M060, M063, M069, M050, M052, M051, M353
No	0	
**Peptic ulcer disease**		
Yes	1	K25, K26, K27, K28
No	0	
**Diabetes**		
Yes, no complications	1	E109, E119, E139, E149, E101, E111, E131, E141, E105, E115, E135, E145
Yes, with chronic complications	2	E102, E112, E132, E142, E103, E113, E133, E143, E104, E114, E134, E144
No	0	
**Hemiplegia or paraplegia**		
Yes	2	G041, G81, G820, G821, G822
No	0	
**Renal disease**		
Yes	2	N03, N052, N053, N054, N055, N056, N072, N073, N074, N01, N18, N19, N25
No	0	
**Malignancy**		
Localized solid tumor	2	C0, C1, C2, C3, C40, C41, C43, C45, C46, C47, C48, C49, C5, C6, C70, C71, C72, C73, C74, C75, C76, C883, C887, C889, C900, C901, C96
Metastatic solid tumor	6	C77, C78, C79, C80
No	0	
**Leukemia**		
Yes	2	C91, C92, C93, C94, C95
No	0	
**Lymphoma**		
Yes	2	C81, C82, C83, C84, C85, C86, C884
No	0	
**AIDS**		
Yes	6	B20, B21, B22, B23, B24
No	0	
**Liver disease**		
Mild liver disease	1	K702, K703, K73, K717, K740, K742, K746, K743, K744, K745
Moderate or severe liver disease	3	K729, K766, K767, K721
No	0	

ICD-10: international classification of disease, 10th revision.

Supplementary Table 4: Characteristics of COVID-19-positive and negative patients.

Covariant		Total population (n=75057)	COVID-19-negative participants (n=58281)	COVID-19-positive participants (n=16776)	p value
**Sex (%)**	Female/Male	53.5/46.5	53.7/46.3	52.8/47.2	0.054
**Age**	Mean(SD)	65.5(8.3)	65.8(8.2)	64.6(8.6)	**<0.001**
	Median[Min,Max]	65.4[49.4, 85.2]	65.8[49.4, 85.2]	63.7[49.5, 82.9]	
**TDI**	Mean(SD)	-0.9(3.3)	-0.9(3.3)	-0.7(3.3)	**<0.001**
	Median[Min,Max]	-1.7[-6.3, 10.8]	-1.8[-6.3, 10.8]	-1.5[-6.3, 10.6]	
**Ethnicity (%)**	White/Non-white	93/7	93.8/6.2	90/10	**<0.001**
	Missing	N=440	N=339	N=101	
**BMI (%)**	Normal	29.1	30.1	25.5	**<0.001**
	Obese	28.2	27.2	31.8	
	Overweight	42.2	42.2	42.4	
	Underweight	0.5	0.5	0.4	
	Missing	N=548	N=404	N=144	
**Smoking status (%)**	Never	53.4	53.6	52.9	0.059
	Current	11.8	11.9	11.6	
	Previous	34.7	34.5	35.5	
	Missing	N=519	N=410	N=109	
**CCI score**	Mean(SD)	1.3(2.1)	1.3(2.1)	1.2(2.2)	**0.003**
	Median[Min,Max]	0[0, 19]	0[0, 19]	0[0, 19]	

COVID-19: corona virus disease 2019; TDI: Townsend deprivation index; BMI: body mass index; SD: standard deviation; CCI: Charlson Comorbidity index. Bold indicates p values less than 0.05.

Supplementary Table 5: The outcome-wide association analyses evaluating the hazards ratio (HR) for comorbidities (defined by three-digit ICD-10 codes) of COVID-19-positive participants compared to matched COVID-19-negative individuals.

ICD-10 code	COVID-19-negative		COVID-19-positive		HR(95%CI)	p value	ICD-10 disease	Category
	No. negative participants	No. case	No. positive participants	No. case				
A099	55405	52	15963	105	7.38(5.25-10.38)	<0.001	Gastroenteritis and colitis of unspecified origin	Gastroenteritis and colitis
A415	57991	4	16654	13	12.4(4.04-38.06)	<0.001	Septicaemia due to other Gram-negative organisms	Septicaemia
A419	56948	28	16261	55	8.04(5-12.91)	<0.001	Septicaemia, unspecified	
B370	57968	6	16634	34	21.48(9.01-51.22)	<0.001	Candidal stomatitis	Infectious diseases
B956	57621	12	16585	11	3.19(1.4-7.27)	0.006	Staphylococcus aureus as the cause of diseases classified to other chapters	
B962	57180	18	16338	27	6.27(3.38-11.66)	<0.001	Escherichia coli [E. coli] as the cause of diseases classified to other chapters	
B968	57102	8	16379	23	11.62(4.97-27.15)	<0.001	Other specified bacterial agents as the cause of diseases classified to other chapters	
D509	55644	45	16085	24	2.18(1.3-3.66)	0.003	Iron deficiency anaemia, unspecified	Blood cell disease
D649	54425	63	15664	49	2.88(1.96-4.22)	<0.001	Anaemia, unspecified	
D696	57866	9	16635	26	10.77(5.04-23.02)	<0.001	Thrombocytopenia, unspecified	
E119	52643	66	14897	76	4.24(2.98-6.01)	<0.001	Non-insulin-dependent diabetes mellitus with renal complications	Diabetes mellitus
E162	57822	16	16595	36	8.25(4.57-14.89)	<0.001	Hypoglycaemia, unspecified	Hypoglycaemia
E538	57789	9	16571	14	6.74(2.83-16.08)	<0.001	Deficiency of other specified B group vitamins	Vitamin deficiency
E559	57402	27	16484	45	6.36(3.89-10.39)	<0.001	Vitamin D deficiency, unspecified	
E668	57408	16	16520	15	3.46(1.68-7.11)	<0.001	Other obesity	Obesity
E669	52782	114	15132	88	2.7(2.02-3.61)	<0.001	Obesity, unspecified	
E780	48823	119	14049	92	3.21(2.38-4.32)	<0.001	Pure hypercholesterolaemia	Hypercholesterolaemia
E833	58055	14	16685	32	10.28(5.29-19.97)	<0.001	Disorders of phosphorus metabolism	Electrolyte imbalance
E834	57900	25	16617	32	5.39(3.13-9.3)	<0.001	Disorders of magnesium metabolism	
E835	57776	23	16567	30	4.84(2.81-8.36)	<0.001	Disorders of calcium metabolism	
E86	57024	35	16261	129	15.33(10.35-22.7)	<0.001	Volume depletion	
E870	58153	6	16656	55	35.28(15.18-81.97)	<0.001	Hyperosmolality and hypernatraemia	
E871	57230	31	16399	73	9.9(6.36-15.4)	<0.001	Hypo-osmolality and hyponatraemia	
E872	57871	22	16605	44	7.62(4.52-12.84)	<0.001	Acidosis	
E873	58200	3	16716	23	27.26(8.16-91.09)	<0.001	Alkalosis	
E875	57860	15	16594	38	9.39(5.15-17.1)	<0.001	Hyperkalaemia	
E876	57344	37	16394	82	8.97(5.98-13.46)	<0.001	Hypokalaemia	
E877	57927	23	16609	20	3.31(1.8-6.09)	<0.001	Fluid overload	
F019	58143	5	16598	16	12.64(4.63-34.51)	<0.001	Vascular dementia, unspecified	Dementia
F03	57935	15	16433	28	8.77(4.46-17.27)	<0.001	Unspecified dementia	
F050	58191	4	16679	27	25.88(9.05-74)	<0.001	Delirium not superimposed on dementia, so described	Delirium
F051	58186	7	16637	33	21.56(9.03-51.47)	<0.001	Delirium superimposed on dementia	
F058	58200	4	16702	21	20.78(7.13-60.55)	<0.001	Other delirium	
F059	57744	23	16411	86	15.31(9.49-24.7)	<0.001	Delirium, unspecified	
F171	54579	34	15855	19	2.09(1.16-3.77)	0.014	Harmful use due to use of tobacco	Mental disease
F329	53468	67	15408	48	2.44(1.66-3.58)	<0.001	Depressive episode, unspecified	
F419	55448	80	15979	46	2.02(1.39-2.93)	<0.001	Anxiety disorder, unspecified	
G473	56518	18	16336	17	3.22(1.63-6.35)	<0.001	Sleep apnoea	Sleep apnoea
H919	56811	34	16385	17	1.89(1.05-3.42)	0.034	Hearing loss, unspecified	Hearing loss
I10	38523	218	11404	142	2.69(2.09-3.45)	<0.001	Essential (primary) hypertension	Hypertension
I252	55533	45	15993	22	2.02(1.19-3.43)	0.009	Old myocardial infarction	Chronic ischaemic heart disease
I259	54256	66	15673	48	2.64(1.8-3.86)	<0.001	Chronic ischaemic heart disease, unspecified	
I269	57289	43	16496	112	11.27(7.74-16.43)	<0.001	Pulmonary embolism without mention of acute cor pulmonale	Pulmonary embolism
I340	57416	22	16560	16	2.83(1.47-5.43)	0.002	Mitral (valve) insufficiency	Other heart disease
I350	57553	14	16565	14	4(1.88-8.52)	<0.001	Aortic (valve) stenosis	
I447	57569	21	16559	17	3.05(1.59-5.84)	<0.001	Left bundle-branch block, unspecified	
I451	57495	31	16532	17	2.52(1.36-4.68)	0.003	Other and unspecified right bundle-branch block	
I480	57145	35	16495	22	2.34(1.37-4.02)	0.002	Paroxysmal atrial fibrillation	
I489	54930	100	15785	96	4.07(3.03-5.47)	<0.001	Atrial fibrillation and atrial flutter, unspecified	
I500	57442	24	16411	27	4.96(2.81-8.79)	<0.001	Congestive heart failure	
I509	57394	32	16447	30	3.88(2.33-6.47)	<0.001	Heart failure, unspecified	
I517	56868	47	16329	36	3.2(2.03-5.03)	<0.001	Cardiomegaly	
I518	57389	29	16458	22	2.84(1.62-4.97)	<0.001	Other ill-defined heart diseases	
I678	57595	39	16434	19	1.89(1.08-3.3)	0.026	Other specified cerebrovascular diseases	Cerebrovascular diseases
I739	57447	12	16511	12	3.71(1.63-8.45)	0.002	Peripheral vascular disease, unspecified	Vascular disease
I802	57544	11	16544	14	5.47(2.43-12.32)	<0.001	Phlebitis and thrombophlebitis of other deep vessels of lower extremities	
I951	57466	26	16424	28	4.79(2.74-8.39)	<0.001	Orthostatic hypotension	Hypotension
I959	56751	38	16256	85	8.53(5.77-12.62)	<0.001	Hypotension, unspecified	
J128	58256	3	15896	1016	3714.24(522.68-26393.82)	<0.001	Other viral pneumonia	Lower respiratory infection
J181	56277	53	16023	58	4.4(2.99-6.47)	<0.001	Lobar pneumonia, unspecified	
J189	56903	29	16218	67	9.63(6.07-15.26)	<0.001	Pneumonia, unspecified	
J22	55911	30	15953	39	4.94(3.04-8.04)	<0.001	Unspecified acute lower respiratory infection	
J690	58042	7	16633	23	12.08(5.17-28.22)	<0.001	Pneumonitis due to food and vomit	
J439	57683	16	16576	25	5.82(3.1-10.92)	<0.001	Emphysema, unspecified	COPD/Emphysema
J440	57439	9	16391	70	29.18(14.57-58.45)	<0.001	Chronic obstructive pulmonary disease with acute lower respiratory infection	
J459	51511	67	14821	46	2.44(1.64-3.61)	<0.001	Asthma, unspecified	Asthma
J47	57525	19	16569	21	4.4(2.34-8.27)	<0.001	Bronchiectasis	Other lung disease
J841	57975	10	16658	23	10.88(4.86-24.37)	<0.001	Other interstitial pulmonary diseases with fibrosis	
J981	57085	37	16368	43	4.62(2.94-7.25)	<0.001	Pulmonary collapse	
J90	56560	61	16194	50	3.51(2.37-5.19)	<0.001	Pleural effusion, not elsewhere classified	Pleural effusion
J9600	58246	2	16703	45	81.26(19.69-335.32)	<0.001	Acute respiratory failure; Type I [hypoxic]	Respiratory failure
J9690	58037	8	16497	275	123.04(60.9-248.59)	<0.001	Respiratory failure, unspecified; Type I [hypoxic]	
J9691	58112	7	16684	25	13.34(5.76-30.88)	<0.001	Respiratory failure unspecified; Type II [hypercapnic]	
K219	51996	105	15210	65	2.32(1.68-3.2)	<0.001	Gastro-oesophageal reflux disease without oesophagitis	Gastro-oesophageal reflux disease
K579	55837	63	16110	38	2.54(1.68-3.86)	<0.001	Diverticular disease of intestine, part unspecified, without perforation or abscess	Diverticular disease of intestine

**K590**	54181	67	15477	86	4.81(3.46-6.69)	**<0.001**	Constipation	Fecal abnormalities
**R15**	57641	12	16494	16	4.93(2.32-10.45)	**<0.001**	Faecal incontinence	
**R195**	57483	25	16573	16	2.38(1.27-4.47)	**0.007**	Other fecal abnormalities	
**K760**	57178	43	16440	28	2.17(1.34-3.52)	**0.002**	Fatty (change of) liver, not elsewhere classified	Fatty liver
**L031**	56592	17	16167	12	2.9(1.36-6.22)	**0.006**	Cellulitis of other parts of limb	Cellulitis
**L248**	58188	3	16730	21	27.7(8.26-92.88)	**<0.001**	Irritant contact dermatitis due to other agents	Rash and dermatitis
**R21**	57643	8	16580	20	8.85(3.88-20.14)	**<0.001**	Rash and other nonspecific skin eruption	
**L890**	58211	5	16737	15	14.35(4.76-43.33)	**<0.001**	Stage I decubitus ulcer and pressure area	Decubitus ulcer
**L891**	58086	12	16618	37	14.19(7.05-28.57)	**<0.001**	Stage II decubitus ulcer	
**L899**	58170	4	16689	19	23.27(6.88-78.72)	**<0.001**	Decubitus ulcer and pressure area, unspecified	
**M109**	57206	33	16439	19	2.06(1.17-3.63)	**0.013**	Gout, unspecified	Gout
**M199**	54344	109	15666	83	3.11(2.3-4.19)	**<0.001**	Arthrosis, unspecified	Osteoarthritis
**M549**	56947	20	16404	17	3.46(1.78-6.73)	**<0.001**	Dorsalgia, unspecified	
**M819**	56394	29	16270	26	3.56(2.09-6.07)	**<0.001**	Osteoporosis, unspecified	
**N179**	55619	85	15611	235	11.01(8.49-14.3)	**<0.001**	Acute renal failure, unspecified	Renal failure
**N183**	56635	45	16235	44	3.69(2.42-5.63)	**<0.001**	Chronic kidney disease, stage 3	
**N189**	56918	29	16264	37	5.96(3.54-10.04)	**<0.001**	Chronic renal failure, unspecified	
**N390**	54434	61	15389	68	4.31(3.01-6.15)	**<0.001**	Urinary tract infection, site not specified	Urinary tract infection
**N40**	54705	73	15862	37	2.04(1.35-3.08)	**<0.001**	Hyperplasia of prostate	Hyperplasia of prostate
**R000**	57424	21	16504	29	6.37(3.5-11.61)	**<0.001**	Tachycardia, unspecified	Arrhythmia
**R001**	57184	23	16466	28	4.67(2.65-8.24)	**<0.001**	Bradycardia, unspecified	
**R05**	57096	18	16345	74	15.34(9.04-26.03)	**<0.001**	Cough	Cough
**R060**	56025	51	16046	98	7.42(5.2-10.59)	**<0.001**	Dyspnea	Dyspnea and asphyxia
**R090**	58191	1	16728	18	68.17(9.09-510.94)	**<0.001**	Asphyxia	
**R11**	54538	64	15772	43	2.24(1.51-3.31)	**<0.001**	Nausea and vomiting	Nausea and vomiting
**R263**	58130	9	16652	17	8.12(3.5-18.84)	**<0.001**	Immobility	Abnormalities of gait and mobility
**R268**	57032	30	16172	62	8.54(5.43-13.44)	**<0.001**	Other and unspecified abnormalities of gait and mobility	
**R296**	56792	61	15904	63	4.57(3.15-6.62)	**<0.001**	Tendency to fall involving the nervous and musculoskeletal systems, not elsewhere classified	
**R31**	54888	34	15914	17	1.96(1.07-3.6)	**0.030**	Unspecified haematuria	Urinary abnormality
**R32**	57331	23	16303	40	6.92(4.07-11.76)	**<0.001**	Unspecified urinary incontinence	
**R33**	55785	46	16019	42	3.56(2.31-5.46)	**<0.001**	Retention of urine	
**R410**	57407	32	16297	48	5.83(3.69-9.22)	**<0.001**	Disorientation, unspecified	Disorientation
**R418**	57933	11	16584	15	5.04(2.31-11)	**<0.001**	Other and unspecified symptoms and signs involving cognitive functions and awareness	Other cognitive symptoms
**R451**	58165	6	16703	19	10.99(4.36-27.67)	**<0.001**	Restlessness and agitation	Emotional state symptoms and signs
**R458**	58026	8	16684	15	7.66(3.1-18.9)	**<0.001**	Other symptoms and signs involving emotional state	
**R509**	57020	21	16364	61	10.21(6.21-16.79)	**<0.001**	Fever, unspecified	General symptoms and signs
**R51**	55732	34	16071	19	2.06(1.16-3.67)	**0.014**	Headache	
**R53**	57078	25	16336	71	10.6(6.66-16.86)	**<0.001**	Malaise and fatigue	
**R55**	55477	43	15938	22	2.02(1.19-3.45)	**0.009**	Syncope and collapse	
**R568**	57664	11	16551	10	3.33(1.41-7.87)	**0.006**	Other and unspecified convulsions	
**R600**	57655	14	16547	24	6.46(3.33-12.5)	**<0.001**	Localised oedema	
**R630**	57951	11	16658	19	6.34(3.01-13.35)	**<0.001**	Anorexia	
**R638**	58113	14	16669	27	8.03(4.14-15.57)	**<0.001**	Other symptoms and signs concerning food and fluid intake	
**R798**	56138	49	16176	34	2.39(1.54-3.71)	**<0.001**	Other specified abnormal findings of blood chemistry	Abnormal examing results
**R91**	57506	21	16533	16	2.98(1.54-5.75)	**0.001**	Abnormal findings on diagnostic imaging of lung	
**R945**	57035	31	16364	75	9.37(6.1-14.41)	**<0.001**	Abnormal results of liver function studies	

ICD-10: international classification of disease 10th revision; COVID-19, corona virus disease 2019; CI: confidence interval. Bold indicates p values less than 0.05.

Supplementary Table 6: Adjusted risk of death for COVID-19-positive participants compared with matched COVID-19-negative comparisons.

**All COVID-19 patients compared with matched negative patients**					
**Test result**	**Number**	**Death case**	**Death rate**	**HR(95% CI)**	**P value**
COVID-19-negative	57336	522	0.91%	reference	**<0.001**
COVID-19-positive	16483	1169	7.09%	10.6(9.28-12.11)	
**Mild COVID-19 patients compared with matched negative patients**					
**Test result**	**Number**	**Death case**	**Death rate**	**HR(95% CI)**	**P value**
COVID-19-negative	47462	381	0.80%	reference	**<0.001**
Mild COVID-19	13903	205	1.47%	2.11(1.7-2.6)	
**Severe COVID-19 patients compared with matched negative patients**					
**Test result**	**Number**	**Death case**	**Death rate**	**HR(95% CI)**	**P value**
COVID-19-negative	9874	141	1.43%	reference	**<0.001**
Severe COVID-19	2580	964	37.36%	44.15(34.14-57.11)	

Note: Analyses were adjusted for ethnicity, BMI, smoking status, and Charlson Comorbidity index. COVID-19, corona virus disease 2019; HR: hazards ratio; CI: confidence interval. Bold indicates p values less than 0.05.

Supplementary Table 7: Characteristics of mild (non-hospitalized) and severe (hospitalized) COVID-19 patients.

Covariates		All COVID-19 patients (n=16776)	Mild COVID-19 patients (n=14106)	Severe COVID-19 patients (n=2670)	p value
**Sex (%)**	Female/Male	52.8/47.2	55.2/44.8	40.3/59.7	**<0.001**
**Age**	Mean(SD)	64.6(8.6)	63.5(8.3)	70.2(8)	**<0.001**
	Median[Min,Max]	63.7[49.5, 82.9]	62.2[49.5, 82.7]	72.4[49.7, 82.9]	
**TDI**	Mean(SD)	-0.7(3.3)	-0.8(3.2)	0(3.5)	**<0.001**
	Median[Min,Max]	-1.5[-6.3, 10.6]	-1.6[-6.3, 10.6]	-0.8[-6.3, 10]	
**Ethnicity (%)**	White/Non-white	90/10	90.3/9.7	88.9/11.1	**0.033**
	Missing	N=101	N=75	N=26	
**BMI (%)**	Normal	25.5	27.5	14.6	**<0.001**
	Obese	31.8	29.3	45	
	Overweight	42.4	42.8	40	
	Underweight	0.4	0.4	0.3	
	Missing	N=144	N=95	N=49	
**Smoking status (%)**	Never	52.9	54.9	42.7	**<0.001**
	Current	11.6	11.2	13.4	
	Previous	35.5	33.9	43.9	
	Missing	N=109	N=73	N=36	
**CCI score**	Mean(SD)	1.2(2.2)	0.9(1.8)	2.7(2.9)	**<0.001**
	Median[Min,Max]	0[0, 19]	0[0, 19]	2[0, 18]	

COVID-19: corona virus disease 2019; TDI: Townsend deprivation index; BMI: body mass index; CCI: Charlson Comorbidity index. Bold indicates p values less than 0.05.

Supplementary Table 8: Adjusted hazards ratios (HRs) of clinical comorbidities for severe COVID-19 patients (hospitalized) compared with mild COVID-19 patients (non-hospitalized).

Comorbidity	Mild COVID-19			Severe COVID-19			HR (95% CI)	P
	No. mild participants	No. case	Incidence	No. severe participants	No. case	Incidence		
Gastroenteritis and colitis	13612	13	0.10%	2351	92	3.91%	49.28(26.89-90.31)	**<0.001**
Septicaemia	13815	13	0.09%	2372	53	2.23%	20.84(10.79-40.28)	**<0.001**
Infectious diseases	13470	12	0.09%	2297	70	3.05%	25.37(13.38-48.07)	**<0.001**
Blood cell disease	13036	15	0.12%	2148	63	2.93%	22.7(12.55-41.07)	**<0.001**
Diabetes mellitus	12933	6	0.05%	1964	70	3.56%	112.2(47.96-262.46)	**<0.001**
Hypoglycaemia	14034	8	0.06%	2561	28	1.09%	14.68(6.42-33.56)	**<0.001**
Vitamin deficiency	13801	14	0.10%	2501	41	1.64%	18.9(9.9-36.08)	**<0.001**
Obesity	12930	10	0.08%	2120	81	3.82%	95.18(48.34-187.44)	**<0.001**
Hypercholesterolaemia	12249	16	0.13%	1800	76	4.22%	36.02(20.43-63.53)	**<0.001**
Electrolyte imbalance	13376	37	0.28%	1938	269	13.88%	48.23(33.62-69.2)	**<0.001**
Dementia	13854	7	0.05%	2472	28	1.13%	11.66(4.94-27.56)	**<0.001**
Delirium	13841	19	0.14%	2356	123	5.22%	27.86(16.67-46.56)	**<0.001**
Mental disease	12346	15	0.12%	2055	66	3.21%	43.19(23.54-79.22)	**<0.001**
Sleep apnoea	13825	2	0.01%	2511	15	0.60%	52.82(11.51-242.39)	**<0.001**
Hearing loss	13841	2	0.01%	2544	15	0.59%	36.9(8.37-162.65)	**<0.001**
Hypertension	10317	22	0.21%	1087	120	11.04%	56.82(35.53-90.89)	**<0.001**
Chronic ischaemic heart disease	13288	7	0.05%	2133	37	1.73%	27.48(11.83-63.86)	**<0.001**
Pulmonary embolism	13950	17	0.12%	2546	95	3.73%	33.79(19.72-57.91)	**<0.001**
Other heart disease	12944	27	0.21%	1859	129	6.94%	28.45(18.45-43.87)	**<0.001**
Cerebrovascular diseases	13924	4	0.03%	2510	15	0.60%	14.58(4.54-46.79)	**<0.001**
Vascular disease	13833	6	0.04%	2467	18	0.73%	16.11(5.98-43.45)	**<0.001**
Hypotension	13653	16	0.12%	2316	91	3.93%	31.86(18.3-55.48)	**<0.001**
Lower respiratory infection	13141	41	0.31%	1323	816	61.68%	364.28(264.7-501.32)	**<0.001**
COPD/Emphysema	13923	6	0.04%	2364	66	2.79%	47.24(19.95-111.85)	**<0.001**
Asthma	12667	4	0.03%	2154	42	1.95%	132.33(46.31-378.11)	**<0.001**
Other lung disease	13788	10	0.07%	2317	63	2.72%	38.56(19.22-77.36)	**<0.001**
Pleural effusion	13787	6	0.04%	2407	44	1.83%	40.4(16.62-98.21)	**<0.001**
Respiratory failure	14023	8	0.06%	2335	323	13.83%	305.3(150.45-619.53)	**<0.001**
Gastro-oesophageal reflux disease	12964	20	0.15%	2246	45	2.00%	16.83(9.54-29.7)	**<0.001**
Diverticular disease of intestine	13672	10	0.07%	2438	28	1.15%	16.32(7.56-35.23)	**<0.001**
Fecal abnormalities	13058	20	0.15%	2090	84	4.02%	21(12.57-35.09)	**<0.001**
Fatty liver	13887	4	0.03%	2553	24	0.94%	39.15(12.96-118.24)	**<0.001**
Cellulitis	13733	3	0.02%	2434	9	0.37%	15.09(3.8-59.95)	**<0.001**
Rash and dermatitis	13966	8	0.06%	2574	32	1.24%	21.03(9.26-47.76)	**<0.001**
Decubitus ulcer	13995	8	0.06%	2535	58	2.29%	33.45(15.55-71.95)	**<0.001**
Gout	13911	2	0.01%	2528	17	0.67%	33.93(7.83-147.03)	**<0.001**
Osteoarthritis	12992	23	0.18%	2100	82	3.90%	24.51(14.99-40.08)	**<0.001**
Renal failure	13331	28	0.21%	1860	202	10.86%	44.33(29.44-66.75)	**<0.001**
Urinary tract infection	13243	20	0.15%	2146	48	2.24%	11.46(6.52-20.13)	**<0.001**
Hyperplasia of prostate	13514	4	0.03%	2348	33	1.41%	31.75(11.22-89.87)	**<0.001**
Arrhythmia	13764	6	0.04%	2449	46	1.88%	54.16(22.44-130.71)	**<0.001**
Cough	13882	13	0.09%	2463	61	2.48%	34.73(18.44-65.4)	**<0.001**
Dyspnea and asphyxia	13688	19	0.14%	2324	90	3.87%	37.68(22.39-63.43)	**<0.001**
Nausea and vomiting	13431	16	0.12%	2341	27	1.15%	12.96(6.63-25.3)	**<0.001**
Abnormalities of gait and mobility	13471	19	0.14%	2096	89	4.25%	23.84(14.2-40.04)	**<0.001**
Urinary abnormality	12908	13	0.10%	2088	64	3.07%	24.64(13.19-46.02)	**<0.001**
Disorientation	13863	16	0.12%	2434	32	1.31%	8.63(4.48-16.62)	**<0.001**
Other cognitive symptoms	14003	3	0.02%	2581	12	0.46%	12.01(3.21-44.92)	**<0.001**
Emotional state symptoms and signs	14008	7	0.05%	2607	25	0.96%	17.12(6.98-42.01)	**<0.001**
General symptoms and signs	12540	33	0.26%	1893	130	6.87%	27.12(18.07-40.71)	**<0.001**
Abnormal examing results	13383	16	0.12%	2233	93	4.16%	35.53(20.39-61.88)	**<0.001**

Note: Analyses were adjusted for age, sex, TDI, ethnicity, BMI, smoking status, and CCI. COVID-19: corona virus disease 2019; TDI: Townsend deprivation index; BMI: body mass index; CCI: Charlson Comorbidity index; CI: confidence interval; COPD: chronic obstructive pulmonary disease. Bold indicates p values less than 0.05.

Supplementary Table 9: The adjusted risk of clinical comorbidity in COVID-19-positive participants compared with matched COVID-19-negative participants in females and males, respectively.

Comorbidity	Male								Female							
	COVID-19 negative			COVID-19 positive			HR (95% CI)	P value	COVID-19 negative			COVID-19 positive			HR (95% CI)	P value
	No. negative participants	No. case	Incidence	No. positive participants	No. case	Incidence			No. negative participants	No. case	Incidence	No. positive participants	No. case	Incidence		
Gastroenteritis and colitis	25787	20	0.08%	7536	51	0.68%	53.57(9.28-309.36)	**<0.001**	29618	32	0.11%	8427	54	0.64%	5.55(2.88-10.7)	**<0.001**
Septicaemia	26164	16	0.06%	7578	40	0.53%	18.45(5.18-65.7)	**<0.001**	30580	13	0.04%	8609	26	0.30%	97.91(6.64-1444.38)	**<0.001**
Infectious diseases	25645	21	0.08%	7435	51	0.69%	12.12(4.79-30.67)	**<0.001**	29755	16	0.05%	8332	31	0.37%	9.71(3.27-28.8)	**<0.001**
Blood cell disease	24536	39	0.16%	7210	47	0.65%	5.42(2.73-10.78)	**<0.001**	28004	53	0.19%	7974	31	0.39%	1.57(0.87-2.84)	0.134
Diabetes mellitus	23654	41	0.17%	6762	45	0.67%	3.08(1.37-6.92)	**0.006**	28989	25	0.09%	8135	31	0.38%	4.16(1.55-11.22)	**0.005**
Hypoglycaemia	26742	9	0.03%	7801	17	0.22%	NA	0.993	31080	7	0.02%	8794	19	0.22%	20.37(1.71-243.14)	**0.017**
Vitamin deficiency	26483	14	0.05%	7714	26	0.34%	6.98(2.36-20.62)	**<0.001**	30494	19	0.06%	8588	29	0.34%	8.45(3.17-22.53)	**<0.001**
Obesity	24290	55	0.23%	7098	43	0.61%	2.34(1.1-5)	**0.028**	28147	65	0.23%	7952	48	0.60%	1.63(0.8-3.34)	0.181
Hypercholesterolaemia	21400	66	0.31%	6299	49	0.78%	2.73(1.44-5.19)	**0.002**	27423	53	0.19%	7750	43	0.55%	3.03(1.73-5.29)	**<0.001**
Electrolyte imbalance	25060	69	0.28%	7126	179	2.51%	16.22(9.3-28.27)	**<0.001**	29333	68	0.23%	8188	127	1.55%	9.49(5.82-15.47)	**<0.001**
Dementia	26774	9	0.03%	7701	22	0.29%	8.37(2.51-27.95)	**<0.001**	31062	9	0.03%	8625	13	0.15%	14.03(1.5-130.87)	**0.020**
Delirium	26602	20	0.08%	7580	87	1.15%	18.3(7.65-43.78)	**<0.001**	30959	15	0.05%	8617	55	0.64%	16.07(6.08-42.45)	**<0.001**
Mental disease	23316	47	0.20%	6926	40	0.58%	2.67(1.39-5.13)	**0.003**	26250	71	0.27%	7475	41	0.55%	2(1.17-3.39)	**0.011**
Hypertension	16410	105	0.64%	4966	97	1.95%	2.77(1.74-4.41)	**<0.001**	22113	113	0.51%	6438	45	0.70%	1.45(0.84-2.49)	0.184
Pulmonary embolism	26463	22	0.08%	7766	81	1.04%	32.17(11.08-93.39)	**<0.001**	30826	21	0.07%	8730	31	0.36%	5.22(2.44-11.18)	**<0.001**
Other heart disease	22829	94	0.41%	6691	100	1.49%	3.67(2.37-5.68)	**<0.001**	28722	68	0.24%	8112	56	0.69%	4.35(2.48-7.61)	**<0.001**
Cerebrovascular diseases	26632	21	0.08%	7722	10	0.13%	4.74(1.2-18.77)	**0.027**	30963	18	0.06%	8712	9	0.10%	1.79(0.46-6.97)	0.400
Vascular disease	26045	15	0.06%	7606	15	0.20%	3.53(1.05-11.93)	**0.042**	30689	7	0.02%	8694	9	0.10%	4.11(0.61-27.55)	0.146
Hypotension	25864	34	0.13%	7464	58	0.78%	11.15(5.04-24.67)	**<0.001**	30205	23	0.08%	8505	49	0.58%	12.7(4.96-32.54)	**<0.001**
Lower respiratory infection	24433	49	0.20%	6624	517	7.80%	107.04(48.83-234.66)	**<0.001**	28967	39	0.13%	7840	340	4.34%	40.4(23.03-70.88)	**<0.001**
COPD/Emphysema	26343	8	0.03%	7633	49	0.64%	1732.86(2.07-1448251.4)	**0.030**	30705	9	0.03%	8654	23	0.27%	NA	0.997
Asthma	24307	27	0.11%	7098	18	0.25%	4.94(1.8-13.54)	**0.002**	27204	40	0.15%	7723	28	0.36%	2.49(1.21-5.1)	**0.013**
Other lung disease	25963	30	0.12%	7551	40	0.53%	4.33(2.16-8.7)	**<0.001**	30229	30	0.10%	8554	33	0.39%	6.69(2.83-15.82)	**<0.001**
Pleural effusion	26040	35	0.13%	7563	23	0.30%	2.22(0.98-5.02)	0.055	30520	26	0.09%	8631	27	0.31%	5.91(2.42-14.4)	**<0.001**
Respiratory failure	26773	12	0.04%	7664	223	2.91%	906.77(67.42-12195.04)	**<0.001**	31079	5	0.02%	8694	108	1.24%	64.43(17.57-236.32)	**<0.001**
Gastro-oesophageal reflux disease	24311	55	0.23%	7215	30	0.42%	1.81(0.98-3.35)	0.057	27685	50	0.18%	7995	35	0.44%	3.53(1.82-6.84)	**<0.001**
Diverticular disease of intestine	25947	36	0.14%	7615	16	0.21%	1.69(0.72-3.97)	0.228	29890	27	0.09%	8495	22	0.26%	3.35(1.44-7.78)	**0.005**
Fecal abnormalities	24907	45	0.18%	7195	66	0.92%	7.08(3.7-13.56)	**<0.001**	28191	48	0.17%	7953	38	0.48%	3.71(1.96-7.02)	**<0.001**
Rash and dermatitis	26654	5	0.02%	7792	24	0.31%	33.8(2.8-407.91)	**0.006**	30902	5	0.02%	8748	16	0.18%	NA	0.994
Decubitus ulcer	26825	10	0.04%	7769	40	0.51%	NA	0.990	31119	8	0.03%	8761	26	0.30%	16.91(3.45-82.9)	**<0.001**
Osteoarthritis	24821	53	0.21%	7306	45	0.62%	3.24(1.83-5.73)	**<0.001**	27251	81	0.30%	7786	60	0.77%	2.46(1.5-4.03)	**<0.001**
Renal failure	24612	65	0.26%	7006	140	2.00%	8.06(4.96-13.11)	**<0.001**	29349	49	0.17%	8185	90	1.10%	9.63(5.42-17.12)	**<0.001**
Urinary tract infection	25324	36	0.14%	7300	35	0.48%	8.24(3.29-20.61)	**<0.001**	29110	25	0.09%	8089	33	0.41%	8.52(3.64-19.94)	**<0.001**
Arrhythmia	25942	21	0.08%	7569	31	0.41%	10.02(3.15-31.81)	**<0.001**	30434	21	0.07%	8644	21	0.24%	3.84(1.61-9.18)	**0.002**
Cough	26455	12	0.05%	7694	44	0.57%	31.7(6.78-148.13)	**<0.001**	30641	6	0.02%	8651	30	0.35%	270.52(2.23-32745.97)	**0.022**
Dyspnea and asphyxia	25945	26	0.10%	7541	62	0.82%	20.18(6.9-59.03)	**<0.001**	30014	25	0.08%	8471	47	0.55%	9.54(4.06-22.43)	**<0.001**
Nausea and vomiting	25705	28	0.11%	7524	16	0.21%	2.91(0.92-9.24)	0.070	28833	36	0.12%	8248	27	0.33%	3.1(1.56-6.15)	**0.001**
Abnormalities of gait and mobility	25884	45	0.17%	7278	64	0.88%	4.86(2.71-8.72)	**<0.001**	30038	34	0.11%	8289	44	0.53%	6.23(3.14-12.38)	**<0.001**
Urinary abnormality	23420	49	0.21%	6879	46	0.67%	3.02(1.65-5.54)	**<0.001**	28808	34	0.12%	8117	31	0.38%	8.29(3.37-20.41)	**<0.001**
Disorientation	26507	21	0.08%	7640	32	0.42%	15.81(4.27-58.6)	**<0.001**	30900	11	0.04%	8657	16	0.18%	95.17(2.03-4459.31)	**0.020**
Emotional state symptoms and signs	26807	8	0.03%	7820	19	0.24%	24.02(3.21-179.82)	**0.002**	31111	5	0.02%	8795	13	0.15%	16.54(1.08-252.35)	**0.044**
General symptoms and signs	23479	60	0.26%	6788	96	1.41%	7.19(4.08-12.66)	**<0.001**	27177	64	0.24%	7645	67	0.88%	4.45(2.71-7.31)	**<0.001**
Abnormal examing results	24641	60	0.24%	7221	69	0.96%	7.49(4.16-13.47)	**<0.001**	29718	34	0.11%	8395	40	0.48%	5.63(2.79-11.38)	**<0.001**

Note: Analyses were adjusted for ethnicity, BMI, smoking status and CCI. COVID-19: corona virus disease 2019; BMI: body mass index; CCI: Charlson Comorbidity index; COPD: chronic obstructive pulmonary disease; HR: hazards ratio; CI: confidence interval. Bold indicates p values less than 0.05.

Supplementary Table 10: The adjusted risk of clinical comorbidity in COVID-19-positive participants compared with matched COVID-19-negative participants stratified by BMI.

Comorbidity	Overweight or obesity								Normal or underweight							
	COVID-19-negative			COVID-19-positive			HR (95% CI)	P value	COVID-19-negative			COVID-19-positive			HR (95% CI)	P value
	No. negative participants	No. case	Incidence	No. positive participants	No. case	Incidence			No. negative participants	No. case	Incidence	No. positive participants	No. case	Incidence		
Gastroenteritis and colitis	38088	34	0.09%	11692	97	0.83%	11.68(7-19.49)	**<0.001**	17317	18	0.10%	4271	8	0.19%	925.27(0.01-67614845.2)	0.232
Septicaemia	39028	23	0.06%	11840	53	0.45%	15.56(6.92-35)	**<0.001**	17716	6	0.03%	4347	13	0.30%	NA	0.996
Infectious diseases	37987	28	0.07%	11534	69	0.60%	7.97(4.72-13.46)	**<0.001**	17413	9	0.05%	4233	13	0.31%	NA	0.998
Blood cell disease	35996	68	0.19%	11115	58	0.52%	2.58(1.7-3.91)	**<0.001**	16544	24	0.15%	4069	20	0.49%	1.92(0.65-5.65)	0.237
Diabetes mellitus	35091	61	0.17%	10617	75	0.71%	3.98(2.54-6.22)	**<0.001**	17552	5	0.03%	4280	1	0.02%	NA	1.000
Hypoglycaemia	39811	12	0.03%	12169	28	0.23%	12.01(3.67-39.27)	**<0.001**	18011	4	0.02%	4426	8	0.18%	NA	0.999
Vitamin deficiency	39199	26	0.07%	11966	46	0.38%	6.62(3.66-11.98)	**<0.001**	17778	7	0.04%	4336	9	0.21%	4.08(0.7-23.58)	0.117
Obesity	34514	117	0.34%	10646	88	0.83%	2.56(1.82-3.58)	**<0.001**	17923	3	0.02%	4404	3	0.07%	NA	1.000
Hypercholesterolaemia	32462	85	0.26%	10036	79	0.79%	3.82(2.57-5.69)	**<0.001**	16361	34	0.21%	4013	13	0.32%	3.23(0.99-10.61)	0.053
Electrolyte imbalance	37336	104	0.28%	11197	262	2.34%	10.74(7.92-14.56)	**<0.001**	17057	33	0.19%	4117	44	1.07%	4.74(2.06-10.95)	**<0.001**
Dementia	39845	9	0.02%	11989	28	0.23%	19.88(5.71-69.23)	**<0.001**	17991	9	0.05%	4337	7	0.16%	NA	0.997
Delirium	39624	26	0.07%	11874	120	1.01%	17.33(10.1-29.75)	**<0.001**	17937	9	0.05%	4323	22	0.51%	11.97(1.14-125.88)	**0.039**
Mental disease	33839	79	0.23%	10528	70	0.66%	2.87(1.91-4.31)	**<0.001**	15727	39	0.25%	3873	11	0.28%	0.48(0.12-1.92)	0.298
Sleep apnoea	38558	16	0.04%	11916	16	0.13%	3.26(1.19-8.94)	**0.022**	17960	2	0.01%	4420	1	0.02%	NA	1.000
Hearing loss	39059	24	0.06%	12013	15	0.12%	2.28(1.03-5.03)	**0.041**	17752	10	0.06%	4372	2	0.05%	0.94(0.12-7.51)	0.954
Hypertension	24054	168	0.70%	7788	128	1.64%	3.13(2.25-4.35)	**<0.001**	14469	50	0.35%	3616	14	0.39%	0.43(0.12-1.54)	0.194
Chronic ischaemic heart disease	36118	58	0.16%	11183	39	0.35%	2.39(1.45-3.92)	**<0.001**	17209	11	0.06%	4238	5	0.12%	9.45(0.61-145.12)	0.107
Pulmonary embolism	39376	34	0.09%	12097	96	0.79%	11.57(6.92-19.34)	**<0.001**	17913	9	0.05%	4399	16	0.36%	18.54(1.81-189.71)	**0.014**
Other heart disease	34801	135	0.39%	10687	139	1.30%	4.05(2.99-5.47)	**<0.001**	16750	27	0.16%	4116	17	0.41%	2.21(0.58-8.33)	0.243
Vascular disease	38955	14	0.04%	11940	22	0.18%	8.69(3-25.2)	**<0.001**	17779	8	0.04%	4360	2	0.05%	NA	0.999
Hypotension	38559	39	0.10%	11713	90	0.77%	8.69(5.49-13.75)	**<0.001**	17510	18	0.10%	4256	17	0.40%	6.81(1.38-33.68)	**0.019**
Lower respiratory infection	36491	67	0.18%	10480	748	7.14%	64.13(42.74-96.23)	**<0.001**	16909	21	0.12%	3984	109	2.74%	34.23(9.81-119.4)	**<0.001**
COPD/Emphysema	39302	8	0.02%	11962	61	0.51%	1849.55(8.1-422489.31)	**0.007**	17746	9	0.05%	4325	11	0.25%	NA	0.999
Asthma	35052	45	0.13%	10772	42	0.39%	2.72(1.66-4.45)	**<0.001**	16459	22	0.13%	4049	4	0.10%	0.02(0-1.55)	0.076
Other lung disease	38619	37	0.10%	11805	57	0.48%	5.98(3.55-10.07)	**<0.001**	17573	23	0.13%	4300	16	0.37%	13.48(1.45-125.08)	**0.022**
Pleural effusion	38904	46	0.12%	11859	39	0.33%	4.58(2.61-8.06)	**<0.001**	17656	15	0.08%	4335	11	0.25%	34.65(1.31-919.12)	**0.034**
Respiratory failure	39849	12	0.03%	11987	293	2.44%	146.76(54.9-392.36)	**<0.001**	18003	5	0.03%	4371	38	0.87%	NA	0.997
Gastro-oesophageal reflux disease	35325	78	0.22%	11050	55	0.50%	2.57(1.71-3.85)	**<0.001**	16671	27	0.16%	4160	10	0.24%	1.53(0.36-6.56)	0.565
Diverticular disease of intestine	38185	51	0.13%	11782	33	0.28%	2.63(1.56-4.43)	**<0.001**	17652	12	0.07%	4328	5	0.12%	NA	0.999
Fecal abnormalities	36536	69	0.19%	11075	80	0.72%	3.9(2.66-5.73)	**<0.001**	16562	24	0.14%	4073	24	0.59%	2.56(1.13-5.79)	**0.025**
Fatty liver	39185	39	0.10%	12026	27	0.22%	2.66(1.46-4.85)	**0.001**	17993	4	0.02%	4414	1	0.02%	NA	1.000
Cellulitis	38787	16	0.04%	11820	12	0.10%	3.15(1.3-7.65)	**0.011**	17805	1	0.01%	4347	0	0.00%	NA	1.000
Rash and dermatitis	39638	8	0.02%	12136	34	0.28%	19.7(6.26-62.02)	**<0.001**	17918	2	0.01%	4404	6	0.14%	1.72(0.11-27.88)	0.703
Decubitus ulcer	39933	13	0.03%	12148	51	0.42%	87.23(15.4-494.09)	**<0.001**	18011	5	0.03%	4382	15	0.34%	NA	0.999
Osteoarthritis	35628	90	0.25%	11009	90	0.82%	2.98(2.13-4.17)	**<0.001**	16444	44	0.27%	4083	15	0.37%	1.31(0.51-3.32)	0.573
Renal failure	36663	95	0.26%	11000	198	1.80%	9.06(6.51-12.62)	**<0.001**	17298	19	0.11%	4191	32	0.76%	22.01(2.66-182.27)	**0.004**
Urinary tract infection	37305	48	0.13%	11234	54	0.48%	4.1(2.6-6.48)	**<0.001**	17129	13	0.08%	4155	14	0.34%	5.82(1.17-28.92)	**0.031**
Hyperplasia of prostate	37353	61	0.16%	11571	32	0.28%	2.22(1.3-3.79)	**0.004**	17352	12	0.07%	4291	5	0.12%	NA	1.000
Arrhythmia	38737	31	0.08%	11888	43	0.36%	5.87(3.21-10.71)	**<0.001**	17639	11	0.06%	4325	9	0.21%	4.8(0.56-41.47)	0.154
Cough	39273	15	0.04%	11991	64	0.53%	15.34(7.47-31.51)	**<0.001**	17823	3	0.02%	4354	10	0.23%	3.57(0.66-19.33)	0.139
Dyspnea and asphyxia	38329	41	0.11%	11694	92	0.79%	9.63(5.8-15.99)	**<0.001**	17630	10	0.06%	4318	17	0.39%	7.76(1.59-37.88)	**0.011**
Nausea and vomiting	37477	46	0.12%	11562	35	0.30%	2.8(1.66-4.74)	**<0.001**	17061	18	0.11%	4210	8	0.19%	3.47(0.2-59.06)	0.389
Abnormalities of gait and mobility	38394	58	0.15%	11396	91	0.80%	5.71(3.84-8.47)	**<0.001**	17528	21	0.12%	4171	17	0.41%	5.14(0.9-29.29)	0.065
Urinary abnormality	35623	60	0.17%	10936	62	0.57%	3.67(2.41-5.61)	**<0.001**	16605	23	0.14%	4060	15	0.37%	1.76(0.6-5.15)	0.302
Disorientation	39542	19	0.05%	11959	34	0.28%	12.32(5.22-29.09)	**<0.001**	17865	13	0.07%	4338	14	0.32%	6.89(1.12-42.33)	**0.037**
Other cognitive symptoms	39919	7	0.02%	12183	12	0.10%	18.24(1.89-175.94)	**0.012**	18014	4	0.02%	4401	3	0.07%	NA	1.000
Emotional state symptoms and signs	39902	10	0.03%	12215	22	0.18%	8.2(2.99-22.47)	**<0.001**	18016	3	0.02%	4400	10	0.23%	NA	0.998
General symptoms and signs	34671	88	0.25%	10544	137	1.30%	6.13(4.32-8.71)	**<0.001**	15985	36	0.23%	3889	26	0.67%	1.49(0.46-4.77)	0.506
Abnormal examing results	37204	72	0.19%	11393	85	0.75%	4.58(3.12-6.74)	**<0.001**	17155	22	0.13%	4223	24	0.57%	4.43(1.03-18.99)	**0.045**

Note: Analyses were adjusted for ethnicity, smoking status, and CCI. COVID-19: corona virus disease 2019; BMI: body mass index; CCI: Charlson Comorbidity index; COPD: chronic obstructive pulmonary disease; HR: hazards ratio; CI: confidence interval. Bold indicates p values less than 0.05.

Supplementary Table 11: The adjusted risk of clinical comorbidity in COVID-19-positive participants compared with matched COVID-19-negative participants stratified by smoking status.

Comorbidity	Current/ex-smoker								Never-smoker							
	COVID-19-negative			COVID-19-positive			HR (95% CI)	P value	COVID-19-negative			COVID-19-positive			HR (95% CI)	P value
	No. negative participants	No. case	Incidence	No. positive participants	No. case	Incidence			No. negative participants	No. case	Incidence	No. positive participants	No. case	Incidence		
Gastroenteritis and colitis	25409	26	0.10%	7403	58	0.78%	8.07(3.94-16.57)	**<0.001**	29609	26	0.09%	8459	45	0.53%	5.14(2.56-10.33)	**<0.001**
Septicaemia	26022	16	0.06%	7494	44	0.59%	36.47(8.34-159.4)	**<0.001**	30336	13	0.04%	8589	21	0.24%	11.67(3.04-44.75)	**<0.001**
Infectious diseases	25374	25	0.10%	7267	50	0.69%	7.22(3.65-14.3)	**<0.001**	29652	12	0.04%	8401	30	0.36%	NA	0.998
Blood cell disease	24068	55	0.23%	6993	40	0.57%	2.01(1.09-3.71)	**0.025**	28118	37	0.13%	8098	37	0.46%	3.05(1.57-5.94)	**0.001**
Diabetes mellitus	23779	32	0.13%	6810	38	0.56%	1.84(0.9-3.75)	0.095	28532	34	0.12%	8002	37	0.46%	4.49(1.83-11.06)	**0.001**
Hypoglycaemia	26640	12	0.05%	7733	17	0.22%	14.49(1.5-139.87)	**0.021**	30785	4	0.01%	8754	19	0.22%	NA	0.997
Vitamin deficiency	26210	15	0.06%	7591	23	0.30%	8.71(2.95-25.67)	**<0.001**	30367	18	0.06%	8609	32	0.37%	7.2(3.02-17.17)	**<0.001**
Obesity	23868	59	0.25%	6922	46	0.66%	2.63(1.29-5.35)	**0.008**	28209	61	0.22%	8034	44	0.55%	1.79(0.91-3.55)	0.093
Hypercholesterolaemia	21651	60	0.28%	6280	47	0.75%	3.56(1.89-6.71)	**<0.001**	26855	56	0.21%	7687	43	0.56%	2.74(1.61-4.65)	**<0.001**
Electrolyte imbalance	24786	72	0.29%	7018	177	2.52%	12.75(7.89-20.58)	**<0.001**	29240	64	0.22%	8202	126	1.54%	9.32(5.65-15.36)	**<0.001**
Dementia	26625	9	0.03%	7600	20	0.26%	11.07(2.45-49.94)	**0.002**	30810	8	0.03%	8625	15	0.17%	16.56(2.58-106.43)	**0.003**
Delirium	26458	22	0.08%	7515	89	1.18%	21.98(9.52-50.72)	**<0.001**	30701	13	0.04%	8578	50	0.58%	15.35(5.21-45.28)	**<0.001**
Mental disease	21130	64	0.30%	6323	47	0.74%	2.31(1.37-3.9)	**0.002**	28113	54	0.19%	7993	33	0.41%	3.47(1.73-6.95)	**<0.001**
Hypertension	16671	113	0.68%	5018	68	1.36%	1.32(0.82-2.12)	0.253	21634	99	0.46%	6328	74	1.17%	2.72(1.63-4.55)	**<0.001**
Chronic ischaemic heart disease	23914	38	0.16%	7006	23	0.33%	1.62(0.73-3.62)	0.236	29064	31	0.11%	8324	21	0.25%	2.75(1.07-7.08)	**0.035**
Pulmonary embolism	26372	24	0.09%	7696	72	0.94%	9.86(5.06-19.23)	**<0.001**	30517	19	0.06%	8695	40	0.46%	14.89(5.48-40.47)	**<0.001**
Other heart disease	23244	95	0.41%	6717	92	1.37%	4.21(2.71-6.54)	**<0.001**	27962	65	0.23%	8005	64	0.80%	3.59(2.17-5.93)	**<0.001**
Cerebrovascular diseases	26518	24	0.09%	7653	17	0.22%	3.71(1.41-9.74)	**0.008**	30677	14	0.05%	8680	2	0.02%	1.57(0.25-9.97)	0.634
Vascular disease	25911	16	0.06%	7531	13	0.17%	2.55(0.89-7.27)	0.080	30430	6	0.02%	8662	10	0.12%	12.01(1.31-110.45)	**0.028**
Hypotension	25698	28	0.11%	7377	58	0.79%	14.02(5.91-33.24)	**<0.001**	29981	29	0.10%	8490	49	0.58%	6.45(3.15-13.17)	**<0.001**
Lower respiratory infection	24099	48	0.20%	6509	453	6.96%	56.74(30.89-104.25)	**<0.001**	28944	36	0.12%	7882	398	5.05%	53.63(28.3-101.65)	**<0.001**
COPD/Emphysema	25810	15	0.06%	7420	58	0.78%	18.05(6.69-48.69)	**<0.001**	30843	1	0.00%	8770	14	0.16%	NA	0.997
Asthma	23635	25	0.11%	6905	25	0.36%	2.68(1.24-5.78)	**0.012**	27527	42	0.15%	7829	21	0.27%	1.32(0.64-2.72)	0.450
Other lung disease	25696	32	0.12%	7456	38	0.51%	3.97(1.85-8.49)	**<0.001**	30103	27	0.09%	8550	34	0.40%	7.88(3.37-18.43)	**<0.001**
Pleural effusion	25928	29	0.11%	7493	28	0.37%	5.83(2.5-13.62)	**<0.001**	30238	29	0.10%	8604	21	0.24%	3.1(1.34-7.17)	**0.008**
Respiratory failure	26579	13	0.05%	7588	175	2.31%	115.63(28.94-462.09)	**<0.001**	30866	3	0.01%	8667	152	1.75%	244.27(29.59-2016.41)	**<0.001**
Gastro-oesophageal reflux disease	23666	49	0.21%	6997	36	0.51%	3.38(1.78-6.41)	**<0.001**	27974	55	0.20%	8124	29	0.36%	1.51(0.84-2.71)	0.169
Diverticular disease of intestine	25528	39	0.15%	7468	19	0.25%	2.09(0.97-4.49)	0.058	29921	24	0.08%	8538	19	0.22%	3.19(1.32-7.73)	**0.010**
Fecal abnormalities	24280	50	0.21%	6969	65	0.93%	5.93(3.25-10.83)	**<0.001**	28455	42	0.15%	8090	39	0.48%	2.88(1.65-5.03)	**<0.001**
Rash and dermatitis	26513	6	0.02%	7720	22	0.28%	24.88(3.67-168.8)	**0.001**	30640	4	0.01%	8714	18	0.21%	11.67(2.61-52.17)	**0.001**
Decubitus ulcer	26684	13	0.05%	7692	43	0.56%	59.81(6.58-543.51)	**<0.001**	30852	5	0.02%	8732	23	0.26%	NA	0.997
Gout	26252	16	0.06%	7631	12	0.16%	5.82(1.49-22.67)	**0.011**	30550	17	0.06%	8704	7	0.08%	2.21(0.62-7.85)	0.220
Osteoarthritis	23698	66	0.28%	6924	56	0.81%	3.47(2.08-5.78)	**<0.001**	28025	68	0.24%	8077	48	0.59%	2.18(1.3-3.64)	**0.003**
Renal failure	24545	55	0.22%	6924	139	2.01%	12.03(7.06-20.48)	**<0.001**	29056	58	0.20%	8174	85	1.04%	6.44(3.83-10.84)	**<0.001**
Urinary tract infection	24931	37	0.15%	7096	39	0.55%	3.47(1.91-6.31)	**<0.001**	29149	24	0.08%	8199	28	0.34%	9.1(3.15-26.33)	**<0.001**
Hyperplasia of prostate	24878	39	0.16%	7313	22	0.30%	2.38(1.21-4.71)	**0.012**	29456	33	0.11%	8448	14	0.17%	2.13(0.87-5.24)	0.100
Arrhythmia	25892	20	0.08%	7531	27	0.36%	7.89(3.02-20.62)	**<0.001**	30093	21	0.07%	8581	24	0.28%	4.36(1.86-10.2)	**<0.001**
Cough	26256	9	0.03%	7601	39	0.51%	NA	0.993	30443	9	0.03%	8643	33	0.38%	12.87(3.9-42.47)	**<0.001**
Dyspnea and asphyxia	25687	33	0.13%	7397	60	0.81%	13.34(5.64-31.55)	**<0.001**	29889	17	0.06%	8515	47	0.55%	11.03(4.37-27.83)	**<0.001**
Abnormalities of gait and mobility	25603	45	0.18%	7145	58	0.81%	5.37(2.97-9.72)	**<0.001**	29945	34	0.11%	8332	50	0.60%	6.46(3.13-13.31)	**<0.001**
Urinary abnormality	23741	40	0.17%	6887	39	0.57%	2.89(1.59-5.24)	**<0.001**	28137	43	0.15%	8016	38	0.47%	3.66(2.03-6.58)	**<0.001**
Disorientation	26415	22	0.08%	7577	25	0.33%	3.29(1.47-7.36)	**0.004**	30594	10	0.03%	8624	22	0.26%	5.74(1.86-17.68)	**0.002**
Other cognitive symptoms	26704	5	0.02%	7732	11	0.14%	5(1.03-24.17)	**0.045**	30826	6	0.02%	8744	4	0.05%	NA	0.999
Emotional state symptoms and signs	26670	6	0.02%	7756	18	0.23%	41.61(2.98-582)	**0.006**	30841	7	0.02%	8755	13	0.15%	18.61(1.71-202.15)	**0.016**
General symptoms and signs	23167	68	0.29%	6659	87	1.31%	4.71(2.93-7.57)	**<0.001**	27156	54	0.20%	7692	74	0.96%	5.16(2.96-8.99)	**<0.001**
Abnormal examing results	24784	60	0.24%	7171	54	0.75%	3.19(1.92-5.32)	**<0.001**	29190	34	0.12%	8347	54	0.65%	6.72(3.49-12.95)	**<0.001**

Note: Analyses were adjusted for ethnicity, BMI and CCI. COVID-19: corona virus disease 2019; BMI: body mass index; CCI: Charlson Comorbidity index; COPD: chronic obstructive pulmonary disease; HR: hazards ratio; CI: confidence interval. Bold indicates p values less than 0.05.

Supplementary Table 12: The adjusted risk of clinical comorbidity in COVID-19-positive participants compared with matched COVID-19-negative participants stratified by CCI score.

Comorbidity	CCI high								CCI low							
	COVID-19-negative			COVID-19-positive			HR (95% CI)	P value	COVID-19-negative			COVID-19-positive			HR (95% CI)	P value
	No. negative participants	No. case	Incidence	No. positive participants	No. case	Incidence			No. negative participants	No. case	Incidence	No. positive participants	No. case	Incidence		
Gastroenteritis and colitis	24329	36	0.15%	6440	73	1.13%	7.89(4.26-14.61)	**<0.001**	31076	16	0.05%	9523	32	0.34%	4.92(2.05-11.81)	**<0.001**
Septicaemia	25099	23	0.09%	6510	47	0.72%	25.45(7.77-83.32)	**<0.001**	31645	6	0.02%	9677	19	0.20%	22.26(2.84-174.35)	**0.003**
Infectious diseases	24196	31	0.13%	6232	61	0.98%	10.96(5.38-22.3)	**<0.001**	31204	6	0.02%	9535	21	0.22%	7.25(1.66-31.58)	**0.008**
Blood cell disease	22106	68	0.31%	5834	56	0.96%	3.1(1.89-5.1)	**<0.001**	30434	24	0.08%	9350	22	0.24%	5.55(2.25-13.72)	**<0.001**
Diabetes mellitus	20835	34	0.16%	5183	25	0.48%	2.25(0.79-6.43)	0.131	31808	32	0.10%	9714	51	0.53%	5.36(2.51-11.45)	**<0.001**
Hypoglycaemia	26049	14	0.05%	6886	35	0.51%	12.04(4.3-33.69)	**<0.001**	31773	2	0.01%	9709	1	0.01%	NA	1.000
Vitamin deficiency	25464	20	0.08%	6663	41	0.62%	8.85(3.89-20.17)	**<0.001**	31513	13	0.04%	9639	14	0.15%	5.17(1.51-17.72)	**0.009**
Obesity	22130	60	0.27%	5771	45	0.78%	2.49(1.17-5.32)	**0.018**	30307	60	0.20%	9279	46	0.50%	0.78(0.4-1.53)	0.473
Hypercholesterolaemia	19147	57	0.30%	4923	37	0.75%	3.07(1.58-5.98)	**<0.001**	29676	62	0.21%	9126	55	0.60%	3.02(1.86-4.92)	**<0.001**
Electrolyte imbalance	23199	98	0.42%	5768	214	3.71%	9.75(6.74-14.11)	**<0.001**	31194	39	0.13%	9546	92	0.96%	7.43(4.35-12.7)	**<0.001**
Dementia	26074	11	0.04%	6651	27	0.41%	16.71(3.83-73.01)	**<0.001**	31762	7	0.02%	9675	8	0.08%	22.8(1.06-491.66)	**0.046**
Delirium	25840	26	0.10%	6535	105	1.61%	16.94(8.48-33.84)	**<0.001**	31721	9	0.03%	9662	37	0.38%	26.11(5.66-120.36)	**<0.001**
Mental disease	20361	56	0.28%	5414	47	0.87%	3.36(1.72-6.57)	**<0.001**	29205	62	0.21%	8987	34	0.38%	2.05(1.18-3.57)	**0.011**
Sleep apnoea	25134	7	0.03%	6734	13	0.19%	10.15(0.99-104.08)	0.051	31384	11	0.04%	9602	4	0.04%	NA	0.998
Hearing loss	25385	21	0.08%	6749	15	0.22%	3.97(1.46-10.81)	**0.007**	31426	13	0.04%	9636	2	0.02%	0.24(0.03-2.11)	0.197
Hypertension	12327	79	0.64%	3145	51	1.62%	2.98(1.37-6.5)	**0.006**	26196	139	0.53%	8259	91	1.10%	2.31(1.58-3.38)	**<0.001**
Chronic ischaemic heart disease	21932	50	0.23%	5797	32	0.55%	3.13(1.52-6.46)	**0.002**	31395	19	0.06%	9624	12	0.12%	1.63(0.63-4.2)	0.311
Pulmonary embolism	25734	27	0.10%	6834	67	0.98%	15.09(6.65-34.23)	**<0.001**	31555	16	0.05%	9662	45	0.47%	11.84(4.35-32.23)	**<0.001**
Other heart disease	20935	92	0.44%	5345	91	1.70%	5.6(3.29-9.54)	**<0.001**	30616	70	0.23%	9458	65	0.69%	4.05(2.44-6.74)	**<0.001**
Cerebrovascular diseases	25787	20	0.08%	6720	15	0.22%	18.06(1.87-174.11)	**0.012**	31808	19	0.06%	9714	4	0.04%	0.42(0.08-2.21)	0.306
Vascular disease	25117	19	0.08%	6645	16	0.24%	5.96(1.97-18.08)	**0.002**	31617	3	0.01%	9655	8	0.08%	NA	0.998
Hypotension	24620	39	0.16%	6357	81	1.27%	9.94(5.38-18.38)	**<0.001**	31449	18	0.06%	9612	26	0.27%	6.05(2.16-16.96)	**<0.001**
Lower respiratory infection	22404	62	0.28%	5157	501	9.71%	41.85(25.53-68.6)	**<0.001**	30996	26	0.08%	9307	356	3.83%	47.17(25.78-86.3)	**<0.001**
COPD/Emphysema	25240	12	0.05%	6573	60	0.91%	40.12(8.08-199.06)	**<0.001**	31808	5	0.02%	9714	12	0.12%	NA	0.998
Asthma	19703	21	0.11%	5107	16	0.31%	3.42(1.19-9.77)	**0.022**	31808	46	0.14%	9714	30	0.31%	1.71(0.96-3.04)	0.070
Other lung disease	24585	42	0.17%	6447	50	0.78%	6.08(3.18-11.63)	**<0.001**	31607	18	0.06%	9658	23	0.24%	7.38(2.62-20.8)	**<0.001**
Pleural effusion	24993	43	0.17%	6552	33	0.50%	3.9(1.98-7.67)	**<0.001**	31567	18	0.06%	9642	17	0.18%	9.09(2.58-31.95)	**<0.001**
Respiratory failure	26075	12	0.05%	6704	204	3.04%	146.96(31.86-677.84)	**<0.001**	31777	5	0.02%	9654	127	1.32%	143.95(17.63-1175.14)	**<0.001**
Gastro-oesophageal reflux disease	22069	55	0.25%	5966	39	0.65%	4.83(2.42-9.66)	**<0.001**	29927	50	0.17%	9244	26	0.28%	1.46(0.81-2.63)	0.208
Diverticular disease of intestine	24660	40	0.16%	6542	28	0.43%	3.33(1.74-6.35)	**<0.001**	31177	23	0.07%	9568	10	0.10%	4.62(1.15-18.64)	**0.031**
Fecal abnormalities	22845	60	0.26%	5834	70	1.20%	5.42(3.22-9.12)	**<0.001**	30253	33	0.11%	9314	34	0.37%	2.75(1.51-5.03)	**0.001**
Fatty liver	25585	25	0.10%	6794	17	0.25%	4.34(0.98-19.2)	0.053	31593	18	0.06%	9646	11	0.11%	1.01(0.37-2.76)	0.984
Cellulitis	25211	11	0.04%	6582	7	0.11%	1.22(0.31-4.88)	0.778	31381	6	0.02%	9585	5	0.05%	2.01(0.38-10.54)	0.409
Rash and dermatitis	25899	8	0.03%	6867	28	0.41%	25.01(5.12-122.17)	**<0.001**	31657	2	0.01%	9673	12	0.12%	NA	0.996
Decubitus ulcer	26168	18	0.07%	6836	51	0.75%	34.44(9.61-123.41)	**<0.001**	31776	0	0.00%	9694	15	0.15%	NA	0.999
Gout	25607	21	0.08%	6790	13	0.19%	2.86(0.97-8.43)	0.057	31599	12	0.04%	9649	6	0.06%	1.63(0.28-9.31)	0.584
Osteoarthritis	21836	71	0.33%	5734	73	1.27%	4.92(2.97-8.15)	**<0.001**	30236	63	0.21%	9358	32	0.34%	1.45(0.83-2.53)	0.191
Renal failure	22460	77	0.34%	5576	146	2.62%	9.48(5.78-15.55)	**<0.001**	31501	37	0.12%	9615	84	0.87%	7.89(4.38-14.2)	**<0.001**
Urinary tract infection	23587	43	0.18%	5947	50	0.84%	5.73(3.11-10.54)	**<0.001**	30847	18	0.06%	9442	18	0.19%	1.9(0.72-5)	0.194
Hyperplasia of prostate	23953	45	0.19%	6394	19	0.30%	1.53(0.74-3.2)	0.253	30752	28	0.09%	9468	18	0.19%	1.56(0.61-4)	0.352
Arrhythmia	24997	30	0.12%	6606	27	0.41%	7.52(2.77-20.39)	**<0.001**	31379	12	0.04%	9607	25	0.26%	17.66(3.95-79)	**<0.001**
Cough	25543	14	0.05%	6720	38	0.57%	11.19(4.16-30.11)	**<0.001**	31553	4	0.01%	9625	36	0.37%	NA	0.996
Dyspnea and asphyxia	24647	40	0.16%	6437	63	0.98%	6.17(3.43-11.08)	**<0.001**	31312	11	0.04%	9575	46	0.48%	327.23(8.85-12101.73)	**0.002**
Nausea and vomiting	23781	36	0.15%	6325	29	0.46%	5.07(2.07-12.41)	**<0.001**	30757	28	0.09%	9447	14	0.15%	1.28(0.55-2.96)	0.571
Abnormalities of gait and mobility	24469	50	0.20%	5969	74	1.24%	7.63(4.24-13.71)	**<0.001**	31453	29	0.09%	9598	34	0.35%	3.58(1.82-7.06)	**<0.001**
Urinary abnormality	22333	46	0.21%	5765	56	0.97%	7.23(3.83-13.62)	**<0.001**	29895	37	0.12%	9231	21	0.23%	2.01(0.97-4.18)	0.062
Disorientation	25734	18	0.07%	6634	37	0.56%	17.84(5.84-54.45)	**<0.001**	31673	14	0.04%	9663	11	0.11%	3.11(1.03-9.39)	**0.044**
Other cognitive symptoms	26183	10	0.04%	6882	14	0.20%	6.75(1.76-25.91)	**0.005**	31750	1	0.00%	9702	1	0.01%	NA	1.000
Emotional state symptoms and signs	26176	12	0.05%	6922	25	0.36%	18.02(3.27-99.23)	**<0.001**	31742	1	0.00%	9693	7	0.07%	NA	0.999
General symptoms and signs	21009	76	0.36%	5315	94	1.77%	6.59(3.94-11.02)	**<0.001**	29647	48	0.16%	9118	69	0.76%	5.43(3.13-9.42)	**<0.001**
Abnormal examing results	23421	53	0.23%	6124	66	1.08%	7.2(3.89-13.31)	**<0.001**	30938	41	0.13%	9492	43	0.45%	2.55(1.46-4.44)	**<0.001**

Note: Analyses were adjusted for ethnicity, BMI, and smoking status. COVID-19: corona virus disease 2019; CCI: Charlson Comorbidity index; BMI: body mass index; COPD: chronic obstructive pulmonary disease; HR: hazards ratio; CI: confidence interval. Bold indicates p values less than 0.05.

Supplementary Table 13: The outcome-wide association analyses evaluating the hazards ratio (HR) for sequelae (defined by three-digit ICD-10 codes) of COVID-19-positive participants compared to matched COVID-19-negative participants.

ICD-10 code	COVID-19-negative		COVID-19-positive		HR(95%CI)	P value	ICD-10 disease	Category
	No. negative participants	No. case	No. positive participants	No. case				
**A419**	56822	141	15404	48	1.55(1.1-2.18)	**0.012**	**Septicaemia, unspecified**	**Septicaemia**
**B956**	57490	25	15637	12	2.13(1.03-4.42)	**0.041**	**Staphylococcus aureus as the cause of diseases classified to other chapters**	**Infectious diseases**
**B968**	56984	29	15448	15	2.01(1.06-3.81)	**0.032**	**Other specified bacterial agents as the cause of diseases classified to other chapters**	
**F050**	58064	23	15707	12	2.42(1.17-5)	**0.017**	**Delirium not superimposed on dementia, so described**	**Delirium**
**F051**	58060	37	15677	17	2.05(1.14-3.69)	**0.017**	**Delirium superimposed on dementia**	
**J128**	58129	12	14475	14	6.45(2.69-15.43)	**<0.001**	**Other viral pneumonia**	**Lower respiratory infection**
**J189**	56772	107	15329	46	1.9(1.33-2.73)	**<0.001**	**Pneumonia, unspecified**	
**J841**	57846	30	15704	19	2.6(1.43-4.71)	**0.002**	**Other interstitial pulmonary diseases with fibrosis**	**Interstitial pulmonary diseases**
**J9691**	57987	28	15722	14	2.04(1.06-3.91)	**0.032**	**Respiratory failure unspecified; Type II [hypercapnic]**	**Respiratory failure**
**L891**	57958	64	15671	26	2.02(1.25-3.26)	**0.004**	**Stage II decubitus ulcer**	**Decubitus ulcer**
**R15**	57511	48	15557	20	2.37(1.35-4.16)	**0.003**	**Faecal incontinence**	**Fecal abnormalities**
**R263**	58001	22	15706	15	4.17(1.98-8.8)	**<0.001**	**Immobility**	**Immobility**
**R32**	57190	94	15397	33	1.88(1.23-2.87)	**0.003**	**Unspecified urinary incontinence**	**Urinary abnormality**
**R458**	57897	25	15709	12	2.27(1.1-4.68)	**0.027**	**Other symptoms and signs involving emotional state**	**Emotional state symptoms and signs**

ICD-10: international classification of disease 10th revision; COVID-19, corona virus disease 2019; CI: confidence interval. Bold indicates p values less than 0.05.

Supplementary Table 14: Adjusted hazards ratios (HRs) of sequelae of COVID-19-positive participants compared with matched COVID-19-negative participants.

Sequelae category	Sequelae	COVID-19-negative			COVID-19-positive			HR(95% CI)	P value
		No. negative participants	No. case	Incidence	No. positive participants	No. case	Incidence		
Respiratory system	Lower respiratory infection	58112	14	0.02%	14461	15	0.10%	8.33(2.76-25.08)	**<0.001**
	Interstitial pulmonary disease	57846	30	0.05%	15704	19	0.12%	2.4(1.16-4.95)	**0.018**
Skin	Decubitus ulcer	57958	64	0.11%	15671	26	0.17%	1.96(1.11-3.46)	**0.020**
Symptoms	Fecal abnormalities	57511	48	0.08%	15557	20	0.13%	2.24(1.21-4.17)	**0.011**
	Immobility	58001	22	0.04%	15706	15	0.10%	4.82(1.86-12.51)	**0.001**
	Urinary incontinence	57190	94	0.16%	15397	33	0.21%	1.81(1.15-2.85)	**0.010**

Note: Analyses were adjusted for ethnicity, BMI, smoking status, and CCI score. COVID-19: corona virus disease 2019; BMI: body mass index; CCI: Charlson Comorbidity index; CI: confidence interval; COPD: chronic obstructive pulmonary disease. Bold indicates p values less than 0.05.

Supplementary Figure 1: Flowchart of the study design.

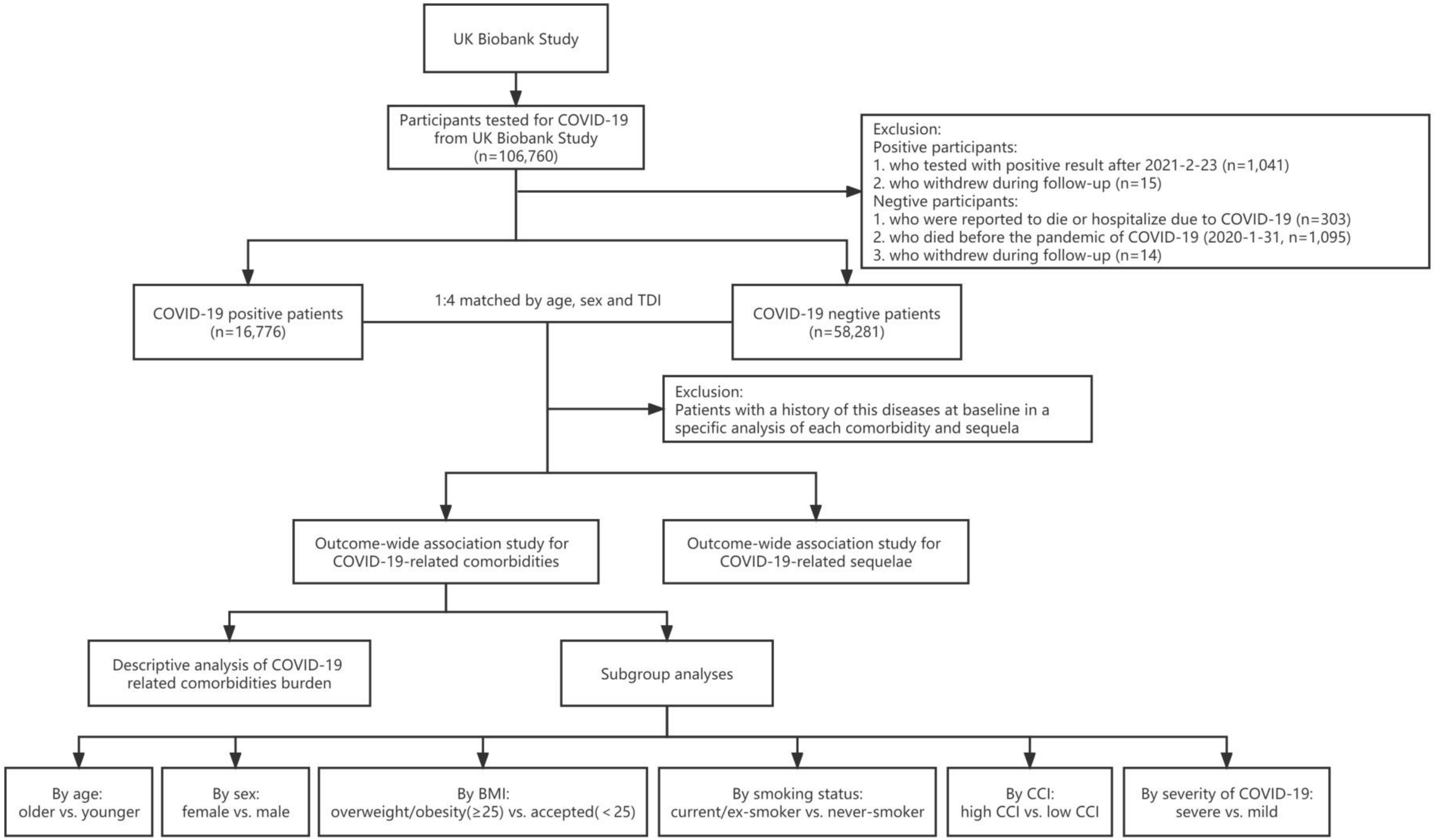

COVID-19: corona virus disease 2019; BMI: body mass index; CCI: Charlson Comorbidity index.

Supplementary Figure 2: The absolute standardized mean difference in all patients and matched patients.

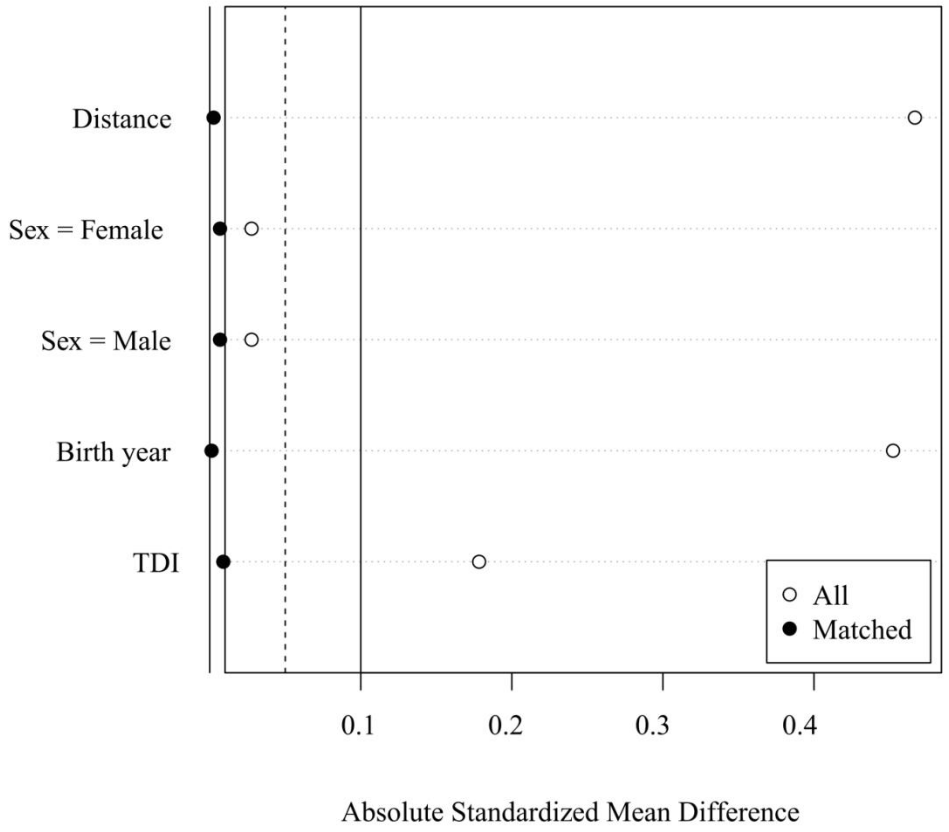

Supplementary Figure 3: The distribution of (A) all, (B) mild (non-hospitalized), and (C) severe (hospitalized) COVID-19 patients with different numbers of comorbidities, respectively.

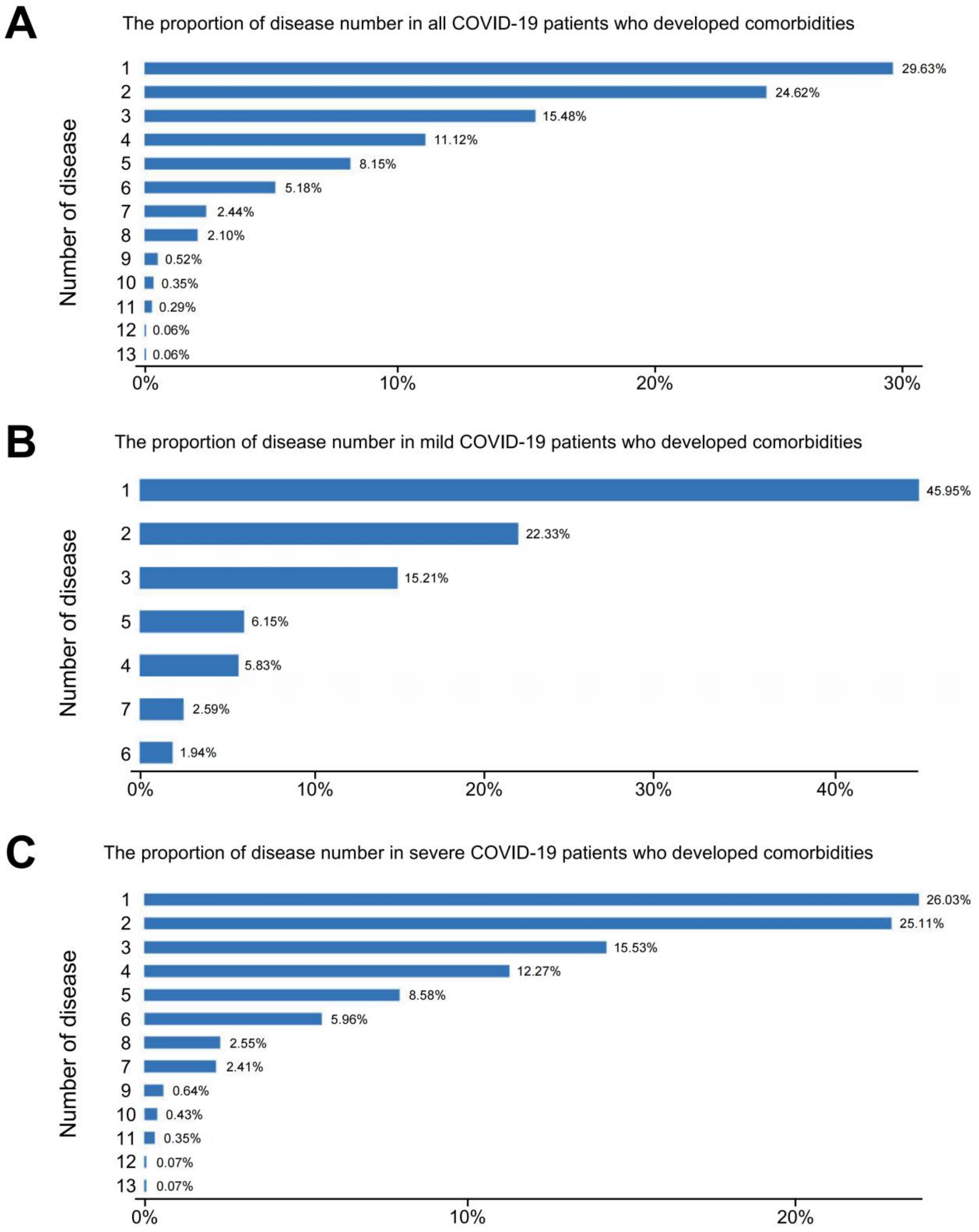

COVID-19: corona virus disease 2019.

Supplementary Figure 4: The proportion of COVID-19-positive participants with different comorbidities, stratified by age, sex, BMI, smoking status, CCI and severity, respectively.

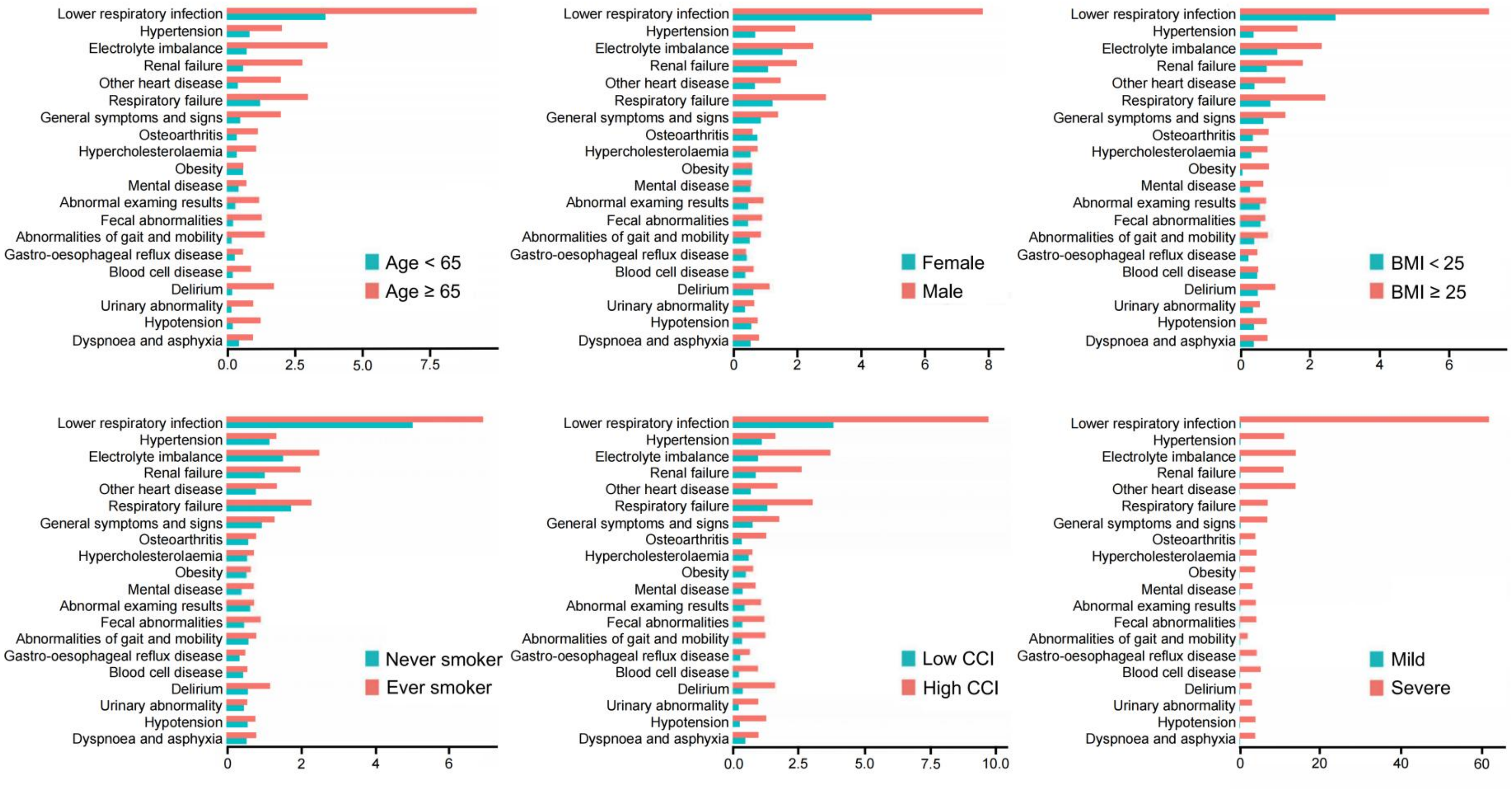

Note: Only the top 20 comorbidities in terms of incidence were shown. COVID-19: corona virus disease 2019; BMI: body mass index; CCI: Charlson Comorbidity index.
